# In the Model Cell Lines of Moderately and Poorly Differentiated Endometrial Carcinoma, Estrogens Can Be Formed *via* the Sulfatase Pathway

**DOI:** 10.3389/fmolb.2021.743403

**Published:** 2021-11-05

**Authors:** Renata Pavlič, Marija Gjorgoska, Eva Hafner, Maša Sinreih, Kristina Gajser, Stefan Poschner, Walter Jäger, Tea Lanišnik Rižner

**Affiliations:** ^1^ Laboratory for Molecular Basis of Hormone-Dependent Diseases and Biomarkers, Institute of Biochemistry and Molecular Genetics, Faculty of Medicine, University of Ljubljana, Ljubljana, Slovenia; ^2^ Department of Pharmaceutical Sciences, University of Vienna, Vienna, Austria

**Keywords:** sulfatase pathway, steroid sulfatase, endometrial cancer, estrone sulfate, estrone sulfate transporters, 17beta-hydroxysteroid dehydrogenase, oxidative metabolism of estrogens, estrogen biosynthesis

## Abstract

Endometrial cancer (EC) is the most common gynecological malignancy in resource-abundant countries. The majority of EC cases are estrogen dependent but the mechanisms of estrogen biosynthesis and oxidative metabolism and estrogen action are not completely understood. Here, we evaluated formation of estrogens in models of moderately and poorly differentiated EC: RL95-2 and KLE cells, respectively. Results revealed high expression of estrone-sulfate (E1-S) transporters (*SLCO1A2, SLCO1B3, SLCO1C1, SLCO3A1, SLC10A6, SLC22A9*), and increased E1-S uptake in KLE *vs* RL95-2 cells. In RL95-2 cells, higher levels of sulfatase and better metabolism of E1-S to E1 were confirmed compared to KLE cells. In KLE cells, disturbed balance in expression of *HSD17B* genes led to enhanced activation of E1 to E2, compared to RL95-2 cells. Additionally, increased *CYP1B1* expression and down-regulation of genes encoding phase II metabolic enzymes: *COMT, NQO1, NQO2*, and *GSTP1* suggested decreased detoxification of carcinogenic metabolites in KLE cells. Results indicate that in model cell lines of moderately and poorly differentiated EC, estrogens can be formed *via* the sulfatase pathway.

## Introduction

Endometrial cancer (EC) is the most frequent gynecological cancer in the developed world (Bray et al., 2018), with a continuing trend for increased incidence over recent decades due to pandemic obesity and increased life expectancy (Lindemann et al., 2010). According to the Bokhman classification, EC histopathology defines two groups. Type I is characterized by well and moderately differentiated endometrioid histology (grades 1, 2), which comprises 70–80% of all cases. In contrast type II, includes clear-cell, serous, or squamous histology, or endometrioid tumors with poorly differentiated histology (grade 3) (Amant et al., 2005; Chiang and Soslow, 2014; Murali et al., 2014; Morice et al., 2016), which comprises 20% of all cases. Tumors that show combined morphological and molecular characteristics of types I and II are also common (Yeramian et al., 2013). In addition to these sporadic cases, about 10% of ECs are hereditary and are associated with Lynch syndrome (Amant et al., 2005). Based on integrated genomic, transcriptomic, and proteomic data, EC is now classified into four molecular subtypes (Kandoth et al., 2013).

EC type I is estrogen-dependent disease, which develops and progresses due to unopposed actions of endogenous and exogenous estrogens on endometrial cells (Inoue, 2001). EC type II is usually considered estrogen independent. However, this has been questioned by studies that have shown no differences in tissue and plasma estrogen concentrations between patients with EC types I and II. This indicates that estrogens also have roles in EC type II (Berstein et al., 2003; Brinton et al., 2016; Wan et al., 2016). EC develops mainly in postmenopausal women, and thus relies on local formation of active estrogens. Locally, estrogens can be formed from the adrenal precursors dehydroepiandrosterone sulfate (DHEA-S) and DHEA, and from circulating estrone sulfate (E1-S) (Rižner et al., 2017). To enter the cells, these sulfated precursors use transporters from the solute carrier gene (*SLC*) superfamily. These genes encode organic anion transporters and organic anion transporting polypeptides (Roth et al., 2012). The efflux of sulfated steroids is *via* ATP-binding cassette (ABC) transporters. Within cells, active estrogens can be synthesized *via* the so-called aromatase and sulfatase pathways by the actions of a series of enzymes that include sulfatase (STS) and aromatase (CYP19A1). The most potent estrogen is estradiol (E2), and it can be formed from DHEA-S or DHEA *via* androstenedione, through the actions of STS, 3β-hydroxysteroid dehydrogenase (HSD3B), aromatase, and reductive 17β-hydroxysteroid dehydrogenases (HSD17B1, HSD17B7, HSD17B12), and also from E1-S through the actions of STS and HSD17B1, HSD17B7, and HSD17B12 (Rižner, 2013). The oxidative enzymes HSD17B2, HSD17B4, HSD17B8, and HSD17B14 catalyze the inactivation of E2 to the less potent estrone (E1). Sulfotransferases SULT2A1 and SULT2B1 maintain the levels of DHEA-S, and SULT1E1 maintains the levels of E1 and estradiol sulfate (E2-S). Estrogens can activate nuclear receptors ERα and ERβ and G protein coupled receptor (GPER), through which they either stimulate cell proliferation (ERα, GPER), induce apoptosis (ERβ) or enhance migration of cancerous cells (GPER) (Paterni et al., 2014; Xu et al., 2019). Additionally, phase I metabolism of estrogens by the CYP enzymes (CYP1A1, CYP1A2, CYP1B1, CYP3A5, CYP3A7) leads to formation of 16α-hydroxyestrogens and 2- and 4-hydroxyestrogens. These catechols can then be further oxidized to quinones, which can bind to DNA and form adducts (Hevir et al., 2011). The formation of estrogen quinones is opposed by phase II metabolism, which includes methylation (catechol-O-methyl transferase; COMT), sulfation (sulfotransferases; SULT1A1, SULT1E1, SULT2B1) and glucuronidation (UDP-glucuronyl transferase). The formation of DNA adducts is prevented by conjugation with glutathione (glutathione S-transferase; GSTP1) and reduction back to catechols by (NAD(P)H quinone dehydrogenases; NQO1, NQO2). Estrogens can stimulate cell proliferation, and can also have genotoxic effects; they can thus act as promoters and initiators of carcinogenesis (Cavalieri and Rogan, 2011; Rižner, 2013; Cavalieri and Rogan, 2016).

The altered uptake of sulfated steroid precursors, the mechanisms of estrogen biosynthesis and oxidative metabolism, and estrogen actions in EC are not completely understood. In the present study we hypothesized that local formation of estrogens *via* the sulfatase pathway has roles in different histological types of endometrial cancer and also in metastatic cancer. We investigated estrogen biosynthesis and metabolism in the RL95-2 and KLE cell lines, as models of moderately and poorly differentiated EC, respectively. The aims were; (i) to examine expression of 20 genes that encode E1-S uptake and efflux transporters, and 31 genes that encode estrogen biosynthetic, phase I and II metabolic enzymes and receptors; (ii) to investigate the metabolism of DHEA-S and E1-S, and to quantify the metabolites they form; and (iii) to measure cell uptake of E1-S in the RL95-2 and KLE cell lines, as representative of moderately and poorly differentiated, metastatic EC.

## Materials and Methods

### Reagents and Chemicals

The standards of E1 (1,3,5 (10)-estratrien-3-ol-17-one) and E2 (1,3,5-estratriene-3,17β-diol) were from Steraloids (Newport, RI, United States), the standards of E1-S (1,3,5 (10)-estratrien-17-one 3-sulfate), E2-S (1,3,5 (10)-estratriene-3,17β-diol sulfate), E2-d_2_ (1,3,5 (10)-estratriene-2,4-d2-3,17β-diol) were from Sigma Aldrich (St. Louis, MO, United States). Methanol and ammonium fluoride (NH_4_F) were from Honeywell International Inc. (Charlotte, NC, United States), and ethanol was from Merck (Darmstadt, Germany). Ultrapure water with a resistivity of 18.2 MΩ × cm was obtained from a Milli-Q water purification system (Merck Millipore, Darmstadt, Germany). The STS inhibitor STX64 was a kind gift from Dr. Barry Potter (Oxford University, Oxford, United Kingdom).

### Model Cell Lines

The RL95-2 cell line (RRID: CVCL_0505) was originally established from a grade 2 moderately differentiated adenosquamous carcinoma of the endometrium from a 65-year-old patient (Way et al., 1983), and it was purchased from American Type Culture Collection (ATCC; CRL-1671; lot 62130010) on October 18, 2017, as passage 125. The RL95-2 cells were cultured in Dulbecco’s modified Eagles’s medium/F12 (D6421), with 10% fetal bovine serum (FBS; F9665), 2.5 mM L-glutamine (G7153), and 5 μg/ml insulin (I9278) (all from Sigma–Aldrich GmbH). The RL95-2 cells were used as passages +8 to +14. Authentication by short tandem repeat (STR) profiling was performed by ATCC.

The KLE cell line (RRID: CVCL_1329) was originally established from a poorly differentiated endometrial carcinoma from a 68-year-old patient (Richardson et al., 1984), and it was purchased from ATCC (CRL-162; lot 70001143) on October 18, 2017, as passage +12. The KLE cells were cultured in Dulbecco’s modified Eagles’s medium/F12 (D6421) supplemented with 10% FBS (F9665) and 2.5 mM L-glutamine (G7153) (all from Sigma–Aldrich GmbH). The KLE cells were used as passages +21 to +27. Authentication by STR profiling was performed by ATCC.

The HIEEC cell line was obtained from Michael A. Fortier (Laval University, Quebec, Canada) on April 4, 2014, as passage 14. The HIEEC cells were grown in RPMI-1640 Medium supplemented with 2 mM L-glutamine and 10% fetal bovine serum (FBS) (all from Sigma-Aldrich, St. Louis, MI, United States). The HIEEC cells were used as passage +7. Cells in passage +8 were authenticated by STR profiling performed by ATCC on March 8, 2018.

These cell lines were all negative for *mycoplasma* infection, according to MycoAlert *mycoplasma* detection kits (Lonza, Basel, Switzerland).

### RNA Isolation

The RL95-2 and KLE cells were cultured four different times independently (n = 4) as two replicates. The total RNA from RL95-2 and KLE cells was isolated and purified using RNA isolation kits (Nucleospin; Macherey-Nagel GmbH & Co. KG, Düren, Germany), according to the manufacturer instructions. The RNA quality was determined using a bioanalyzer (2100: Agilent) and RNA nanokits (RNA 600; Agilent Technologies Inc, Santa Clara, CA, United States). The mean RNA integrity number was 9.00 ± 0.53, which demonstrated that the RNA was of good quality. Samples of the total RNA (4 µg) were reverse transcribed into cDNA (in 40 µg) using cDNA synthesis kits (SuperScript VILO; Invitrogen, Thermo Fisher Scientific, Carlsbad, CA, United States), according to manufacturer instructions. The cDNA samples were stored at −20°C.

### Quantitative PCR

The expression of the genes that encode the enzymes involved in estradiol biosynthesis and oxidative metabolism was examined using quantitative PCR (qPCR) with exon-spanning hydrolysis probes (dye labeled, FAM), as commercially available as ‘Assay on Demand’ (Applied Biosystems, Foster City, CA, United States) (Table 1), or with primers and probes for AKR1C3 that were designed by our group previously (Rižner et al., 2006) (Table 2), using TaqMan Fast Advanced Master Mix and the universal thermocycling parameters recommended by Applied Biosystems (1 cycle of 20 s at 50°C; 1 cycle of 20 s at 95°C; 40 cycles of 1 s at 95°C; 20 s at 60°C). The expression of the genes that encode transporters was examined using SYBR green I master (Roche, Basel, Switzerland) and primers that were designed in our laboratory (Table 3), using the following program: 1 cycle of 5 min at 95°C; 40 cycles of 10 s at 95°C; 10 s at 60°C; and 21 s at 72°C. Quantification was accomplished using a real-time PCR system (ViiA 7; Applied Biosystems, Thermo Fisher Scientific, Waltham, MA, United States). All of the cDNA samples were run on the PCR machine in triplicates, using 0.25 μl cDNA, and the reactions were performed in 384-well plates (MicroAmp Optical; Applied Biosystems, Thermo Fisher Scientific, Waltham, MA, United States), in a reaction volume of 5.0 μl. The PCR amplification efficiency was determined from the slope of the log-linear portion of the calibration curve for each gene investigated, and this was allowed for in the further calculations.

**TABLE 1:** Details for the TaqMan “Assays on Demand” used for the genes investigated in this study.

Gene symbol	Assay ID	Gene name
*COMT*	Hs00241349_m1	Catechol-O-methyltransferase
*CYP19A1*	Hs00240671_m1	Cytochrome P450, family 19, subfamily A
*CYP1A1*	Hs00153120_m1	Cytochrome P450, family 1, subfamily A, polypeptide 1
*CYP1A2*	Hs00167927_m1	Cytochrome P450, family 1, subfamily A, polypeptide 2
*CYP1B1*	Hs00164383_m1	Cytochrome P450, family 1, subfamily B, polypeptide 1
*CYP3A5*	Hs00241417_m1	Cytochrome P450, family 3, subfamily A, polypeptide 5
*CYP3A7*	Hs00426361_m1	Cytochrome P450, family 3, subfamily A, polypeptide 7
*ESR1*	Hs00174860_m1	Estrogen receptor 1 (α)
*ESR2*	Hs00230957_m1	Estrogen receptor 2 (β)
*GPER v2*	Hs00173506_m1	G-protein–coupled estrogen receptor 1 (gene variant 2)
*GPER v3, v4*	Hs01116133_m1	G-protein–coupled estrogen receptor 1 (gene variants 3 and 4)
*GSTP1*	Hs00168310_m1	Glutathione S-transferase pi 1
*HPRT1* *^a^*	Hs99999909_m1	Hypoxanthine phosphoribosyltransferase 1
*HSD17B1*	Hs00166219_g1	Hydroxysteroid (17β) dehydrogenase 1
*HSD17B10*	Hs00189576_m1	Hydroxysteroid (17β) dehydrogenase 10
*HSD17B12*	Hs00275054_m1	Hydroxysteroid (17β) dehydrogenase 12
*HSD17B14*	Hs00212233_m1	Hydroxysteroid (17β) dehydrogenase 14
*HSD17B2*	Hs00157993_m1	Hydroxysteroid (17β) dehydrogenase 2
*HSD17B4*	Hs00264973_m1	Hydroxysteroid (17β) dehydrogenase 4
*HSD17B7*	Hs00367686_m1	Hydroxysteroid (17β) dehydrogenase 7
*HSD17B8*	Hs00367151_m1	Hydroxysteroid (17β) dehydrogenase 8
*HSD3B1*	Hs00426435_m1	Hydroxy-delta-5-steroid dehydrogenase, 3β, and steroid delta-isomerase 1
*HSD3B2*	Hs00605123_m1	Hydroxy-delta-5-steroid dehydrogenase, 3β, and steroid delta-isomerase 2
*NQO1*	Hs00168547_m1	NAD(P)H dehydrogenase, quinone 1
*NQO2*	Hs00168552_m1	NAD(P)H dehydrogenase, quinone 2
*POLR2A* *^a^*	Hs00172187_m1	Polymerase (RNA) II (DNA directed) polypeptide A
*RPLP0* *^a^*	Hs99999902_m1	Ribosomal protein lateral stalk subunit P0
*STS*	Hs00165853_m1	Steroid sulfatase (microsomal), isozyme S
*SULT1A1*	Hs00738644_m1	Sulfotransferase family 1A, member 1
*SULT1E1*	Hs00193690_m1	Sulfotransferase family 1 E, estrogen-preferring, member 1
*SULT2A1*	Hs00234219_m1	Sulfotransferase family, cytosolic, 2A, dehydroepiandrosterone-preferring, member 1
*SULT2B1*	Hs00190268_m1	Sulfotransferase family, cytosolic, 2B, member 1
*UGT2B7*	Hs00426592_m1	UDP glucuronosyltransferase 2 family, polypeptide B7

a Reference genes.

**TABLE 2:** Sequences of the primers and probe for specific amplification of *AKR1C3*.

Gene symbol	Gene name	Primers/probe	Sequence
*AKR1C3*	Aldo–keto reductase family 1, member C3 (17β-hydroxysteroid dehydrogenase type 5)	Forward primers (5′ to 3′)	GTT​GCC​TAT​AGT​GCT​CTG​GGA​TCT
		Reverse primers (5′ to 3′)	GGACTGGGTC CTCCAAGAGG
		Fluorescent MGB-NFQ probe (5′ to 3′)	CACCCATCGTTTGTCTC FAM

**TABLE 3:** Sequences of the primers for SYBR green evaluation of gene expression.

Gene symbol	Gene name	Forward primers (5′ to 3′)	Reverse primers (5′ to 3′)
*ABCC1*	Multidrug-resistance-associated protein 1	GGA​CTC​AGG​AGC​ACA​CGA​AA	ACG​GCG​ATC​CCT​TGT​GAA​AT
*ABCC11*	ATP-binding cassette sub-family C member 11	TCT​CCA​TAT​ATC​CTG​TTA​AT	TAT​AGT​TCT​CCA​GTC​TCT​TG
*ABCC4*	Multidrug-resistance-associated protein 4	AAC​TGC​AAC​TTT​CAC​GGA​TG	AAT​GAC​TTT​TCC​CAG​GCG​TA
*ABCG2*	Broad substrate specificity ATP-binding cassette transporter ABCG2	GGG​TTT​GGA​ACT​GTG​GGT​AG	AGA​TGA​TTC​TGA​CGC​ACA​CC
*HPRT1* *^a^*	Hypoxanthine-guanine phosphoribosyltransferase	CCT​GGC​GTC​GTG​ATT​AGT​C	TGA​GGA​ATA​AAC​ACC​CTT​TCC​A
*POLR2A* *^a^*	DNA-directed RNA polymerase II subunit RPB1	CAA​GTT​CAA​CCA​AGC​CAT​TG	GTGGCAGGTTCTCCAAGG
*RPLP0* *^a^*	60S acidic ribosomal protein P0	AAT​GTG​GGC​TCC​AAG​CAG​AT	TTC​TTG​CCC​ATC​AGC​ACC​AC
*SLC10A6*	Solute carrier family 10 member 6	TAT​GAC​AAC​CTG​TTC​CAC​CG	GAA​TGG​TCA​GGC​ACA​CAA​GG
*SLC22A11*	Solute carrier family 22 member 11	CTC​ACC​TTC​ATC​CTC​CCC​TG	CCA​TTG​TCC​AGC​ATG​TGT​GT
*SLC22A6*	Solute carrier family 22 member 6	CAC​AAG​GAG​GAG​GAA​GAG​GA	ATG​ATG​TGG​TTC​TGG​TGG​GG
*SLC22A7*	Solute carrier family 22 member 7	CCT​CCA​GAG​TCC​AAG​GGT​CT	ATG​CTG​CTC​ACC​CAC​CAA​AT
*SLC22A8*	Solute carrier family 22 member 8	TAC​GCT​GGT​TGG​TCT​TGT​CT	CTC​CCT​CTT​CCT​TCT​TGC​CA
*SLC22A9*	Solute carrier family 22 member 9	CGG​CTA​CCT​ATC​TGA​CCC​CA	TCT​TGA​CGA​CTG​TGC​TTC​CC
*SLC51A*	Organic solute transporter subunit alpha	GCC​CTT​TCC​AAT​ACG​CCT​TC	TCT​GCT​GGG​TCA​TAG​ATG​CC
*SLC51B*	Organic solute transporter subunit beta	GTG​CTG​TCA​GTT​TTC​CTT​CCG	TCA​TGT​GTC​TGG​CTT​AGG​ATG​G
*SLCO1A2*	Solute carrier organic anion transporter family member 1A2	GTT​GGC​ATC​ATT​CTG​TGC​AAA​TGT​T	AAC​GAG​TGT​CAG​TGG​GAG​TTA​TGA​T
*SLCO1B1*	Solute carrier organic anion transporter family member 1B1	CAA​ATT​CTC​ATG​TTT​TAC​TG	GAT​TAT​TTC​CAT​CAT​AGG​TC
*SLCO1B3*	Solute carrier organic anion transporter family member 1B3	TCC​AGT​CAT​TGG​CTT​TGC​AC	TCC​AAC​CCA​ACG​AGA​GTC​CT
*SLCO1C1*	Solute carrier organic anion transporter family member 1C1	CAC​ACA​GAC​TAC​CAA​ACA​CCC	TCA​CCA​TGC​CGA​ACA​GAG​AA
*SLCO2B1*	Solute carrier organic anion transporter family member 2B1	AGA​GCC​CTG​TGT​TCC​ATT​CT	CTC​TTG​CTC​CAG​AAA​TGG​CC
*SLCO3A1*	Solute carrier organic anion transporter family member 3A1	CTA​CGA​CAA​TGT​GGT​CTA​C	TTT​TGA​TGT​AGC​GTT​TAT​AG
*SLCO4A1*	Solute carrier organic anion transporter family member 4A1	ATG​CAC​CAG​TTG​AAG​GAC​AG	AAC​AAG​GTG​GCA​GCT​TCT​GAG
*SLCO4C1*	Solute carrier organic anion transporter family member 4C1	CCAGGAGCCCCAGAAGTC	AAC​TCG​GAC​AGC​GAC​AGT​G

a Reference genes.

For gene expression analysis in RL95-2 and KLE cells, the normalization factor for each sample was calculated based on the geometric mean of the three most stably expressed reference genes (*POLR2A, HPRT1, RPLP0*). The gene expression for each sample was calculated from the crossing-point value (Cq) as E^−Cq^, divided by the normalization factor, and multiplied by 10^13^. The Minimum Information for Publication of Quantitative Real-Time PCR Experiments (MIQE) guidelines were considered in the performance and interpretation of the qPCR reactions (Bustin et al., 2009).

For comparison of RL95-2 and KLE cell lines with HIEEC, expression of *HPRT1* was used as a normalization control. Inter-plate variability in comparison of these two cell lines with HIEEC cells was minimized by the use of relative quatification method and by considering as important only the genes with 10-fold or higher significant differences in expression.

### Western Blotting

Cell lysates were prepared using RIPA Lysis buffer (EMD Millipore Corporation, Temecula, CA, United States) according to the manufacturer instructions. Total protein concentrations were determined using Bradford reagent (Carl Roth GmbH + Co. KG, Karlsruhe, Germany), bovine serum albumin (BSA) as standard, and BioTek (Winooski, VT, United States) PowerWave XS Microplate reader.

Samples of 50 μg protein were separated using SDS PAGE in 10% Tris-glycine gels, and then transferred to poly (vinylidene fluoride) membranes (Millipore, Billerica, MA, United States). For STS detection, the membranes were blocked in 5% non-fat milk overnight and 5% bovine serum albumin in TTBS buffer for 2 h. The membranes were incubated with primary anti-STS antibodies (1:5,000; in TTBS, 5% bovine serum albumin; 2 h at 4°C), which were kindly provided by Dr. Gerhard Schuler (Faculty of Veterinary Medicine, Justus-Liebig-University, Giessen, Germany). The membranes were then incubated for 2 h at 4°C with horseradish-peroxidase-conjugated secondary goat anti-rabbit antibodies (111-035-045, 1:5,000; in TTBS with 2.5% bovine serum albumin; Jackson ImmunoResearch Laboratories Inc, West Grove, PA, United States). For GAPDH detection, the membranes were blocked in 5% non-fat milk at room temperature, and incubated for 1 h in the primary anti-GAPDH antibody (G8795; lot number: 045M4799V; 1:5,000; in TTBS with 1% non-fat milk at room temperature; Sigma-Aldrich, St. Louis, MI, United States). The membranes were then incubated for 1 h at room temperature with the horseradish-peroxidase-conjugated secondary goat anti-mouse antibodies (115-035-062; 1:5,000; in TTBS with 1% nonfat milk; Jackson ImmunoResearch Laboratories Inc, West Grove, PA, United States). SuperSignal West Pico Chemiluminescent Substrate (Thermo Fischer Scientific, Waltham, MA, United States) was used for chemiluminescent detection, with a CCD camera (LAS-4000; Fujifilm, Tokyo, Japan). Differential expression of STS was determined after normalization to GAPDH, using the ImageJ program.

### Steroid Metabolism and Quantification by LC-HRMS

The RL95-2 and KLE cell lines were seeded into six-well plates (92106; TPP, Sigma-Aldrich Chemie GmbH, Deisenhofen, Germany) at 3.5 × 10^6^ cells/well and 3.0 × 10^5^ cells/well, respectively. The next day, when they had reached 70% confluency, the cells were washed twice with Dulbecco’s phosphate-buffered saline (DPBS) and treated with different concentrations of E1, E1-S, DHEA or DHEA-S (10, 100, 500, 1,000 nM), dissolved in dimethylsulfoxide (DMSO) and medium without phenol red and FBS. The final concentration of DMSO was 0.05%. The cells were treated for 48 h, and then the medium was removed and stored at −80°C in glass tubes (6 ml tubes; 986492; Wheaton, VWR, Pennsylvania, United States) until the liquid chromatography–high-resolution mass spectrometry (LC-HRMS) analysis. For normalization purposes, the cells in individual wells were counted using an automated cell counter (TC20; Bio-Rad, CA, United States). Two independent experiments were performed.

A selective and sensitive LC-HRMS assay was used for quantification of steroid precursors and estrogen metabolites (i.e., AD, DHEA, DHEA-S, E1, E1-S, E2, E2-S, E2-glucuronide, estriol, testosterone). This system was validated according to the Q2 (R1) International Conference on Harmonisation guidelines, as described previously (Poschner et al., 2017).

The HPLC system (UltiMate 3000 RSLC-series; Thermo Fisher Scientific, Inc, Waltham, MA, United States) was run with a C18 column (Phenomenex Luna 3 µm C18 (2) 100 Å; 250 × 4.6 mm ID; Phenomenex, Inc, Torrance, CA, United States), and with a C18 guard column (Hypersil BDS; 5 μm, 10 × 4.6 mm ID; Thermo Fisher Scientific, Inc). The column temperature was maintained at 43°C, with 100 µl sample injected. The gradient elution used aqueous ammonium acetate buffer (10 mM, pH 5.0) as solvent A, and acetonitrile as solvent B. The gradient was as follows: 0.0–19.0 min, 25.0–56.3% B; 19.0–19.5 min, 56.3–90.0% B; 19.5–24.0 min, 90.0% B; 24.5–24.5 min, 90.0–25.0% B; 24.5–30.5 min, 25% B. The flow rate was 1.0 ml/min. The HPLC was coupled to a mass spectrometer (maXis HD ESI-Qq-TOF; Bruker Corporation, Bremen, Germany). Full-scan mass spectra were recorded from 150 m*/z* to 500 m*/z*.

### Steroid Metabolism and Quantification by LC-MS/MS

The RL95-2 (passage, +10 to +15) and KLE (passage, +7 to +15) cells were plated into six-well plates at a cell density of 1.0 × 10^6^ cells/well and 1.0 × 10^5^ cells/well, respectively. After 24 h, the cells were washed with DPBS, and serum-free and phenol-red-free culture medium was added. The cells were then incubated with 2.3, 8.5, and 85 nM E1-S (in ethanol; final ethanol concentration, 0.25%). The effects of the STS inhibitor STX64 on E1-S metabolism was evaluated as follows: 30 min before addition of 2.3 nM E1-S, the cells were incubated with 10 nM STX64 (in anhydrous DMSO; final DMSO concentration, 0.25%). After 8, 24, 48, and 72 h of incubation, the cell culture medium was collected in microcentrifuge tubes (Eppendorf, Germany), and stored at −80°C until further processing. Three independent experiments were carried out, each performed in duplicate.

A deuterated internal standard of E2-d_2_ was added to the cell culture medium samples following their thawing at room temperature. The lipophilic fraction containing the analytes of interest was extracted using solid-phase extraction (Strata-X polymer-based columns; Phenomenex, CA, United States). This method involved: column conditioning (1 ml methanol), column equilibration (1 ml water), sample loading, column drying (high vacuum, 10 min), and sample elution (1.5 ml methanol). The solvent was then evaporated off using a vacuum concentrator (Savant SPD 31 DDA-230; Thermo Fisher Scientific, Waltham, MA, United States), and reconstituted in a 100 μl 70% methanol/0.2 mM NH_4_F in water. The samples were stored at −20°C until LC-tandem mass spectrometry (MS/MS) analysis.

An LC-MS/MS system used for detection and quantification of E1, E1-S, E2, and E2-S comprised of a Shimadzu Nexera XR system (Shimadzu Corporation, Kyoto Japan) coupled to a triple quadrupole system (Triple Quad 3 500; AB Sciex Deutchland GmbH, Darmstadt, Germany), operating with the Analyst 1.6 software (AB Sciex Deutchland GmbH, Darmstadt, Germany). Chromatographic separation of the estrogens was performed using a C18 column (Kinetex 2.6 μm XB; 100 × 4.6 mm; Phenomenex, Aschaffenburg, Germany) equipped with a guard column and cartridges (Securityguard C18; 4 × 3.0 mm; Phenomenex, Aschaffenburg, Germany). The mobile phases were 0.2 mM NH_4_F, 5% methanol in water (A) and 0.2 mM NH_4_F in methanol (B). Samples of 25 μl were injected *via* an autosampler (SIL-20 AC XR; Shimadzu Corporation, Kyoto, Japan). The mobile phase flow rate was 0.5 ml/min, and the gradient elution was as follows: 0.0–3.0 min, 70% A; 3.0–8.0 min, 70–4% A; 8.0–8.01 min, 4–70% A; 8.01–15.0 min, 70% A. The column temperature was maintained at 38°C. The MS/MS analysis was performed in negative ion mode with constant electrospray ionization conditions. The source-dependent parameters were as follows: curtain gas, 50; collision gas, 8; ion spray voltage, −4,500 V; source temperature, 600°C; ion source gas 1, 40; ion source gas 2, 80. All transitions were recorded using the scheduled multiple reaction monitoring (MRM) algorithm. The target scan time was set to 1 s, with an MRM detection window of 120 s. The resolution for the first and third quadrupole (Q1, Q3) was set as UNIT, with the pause between the mass ranges set at 5 ms.

The concentration of each steroid was calculated using the internal standard approach. The standard calibration curves were constructed from 12 calibration concentrations prepared in 70% methanol/0.2 mM NH_4_F, to cover the range from 0.01 to 100 ng/ml. An internal standard of E2-d_2_ was added to each sample at a final concentration of 1 ng/ml. The retention times and monitoring transitions for the analytes are given in Supplementary Table S1. The limits of detection and quantification were calculated as 3× and 10× the signal/noise ratio. The limit of detection for E1, E1-S, E2, and E2-S was 1 pg/ml. The limit of quantification for E1 and E1-S was 5 pg/ml, and for E2 and E2-S, 10 pg/ml.

### E1-S Uptake

For analysis of E1-S transport, RL95-2 and KLE cells were seeded into six-well plates at 1.0 × 10^6^ cells/well and 1.2 × 10^5^ cells/well, respectively, in triplicates. Here, RL95-2 cells were between passages +12 and +14, and KLE cells between passages +28 and +32. When the cells reached 60% confluency, full growth medium was replaced with medium without FBS, for 24 h. The cells were then washed twice with warm DPBS (S5652; Sigma-Aldrich, St. Louis, MO, United States) and incubated for 30 min at 37°C in 1.9 ml/well transport buffer (125 mM NaCl, 4.8 mM KCl, 1.2 mM CaCl_2_, 1.2 mM KH_2_PO_4_, 12 mM MgSO_4_, 25 mM MES, 5.6 mM glucose, pH 5.5) with or without 10 µM inhibitor cyclosporine A (CsA, SML1018, Sigma-Aldrich, St. Louis, MI, United States) or 10 µM bromosulphophthalein (BSP, HY-D0217, MedChemExpress, Monmouth Junction, NJ, United States). After 30 min, steroid transport was initiated by addition of 100 µl transport buffer containing [^3^H]E1-S (NET203250UC; Perkin Elmer Inc, MA, United States) to a final concentration of 16 nM, and the cells were incubated for 2, 5, 15, and 30 min at 37°C. The medium was collected and the uptake was stopped with 2 ml ice-cold DPBS. After five washes with DPBS, the cells were lyzed by addition of 300 µ/well 1% Triton X-100, mixed for 30 min at 200 rpm at 4°C, and then frozen at −80°C. After thawing, the lyzed cell samples were collected and centrifuged at 11,000 × *g* for 15 min at 4°C. Finally, 250 µl of the cell lysates was mixed with 1.5 ml scintillation fluid (Quickszint Flow 302; Zinnsser Analytic, Frankfurt, Germany) and the radioactivity was determined in a scintillation counter (MicroBeta TriLux 1450; PerkinElmer, CT, United States). The [^3^H]E1-S concentrations in the individual samples were then back-calculated from the disintegrations per minute (dpm) and the specific isotope activity (49.19 Ci/mmol) with the conversion of 1 Ci = 2.22 × 10^12^ dpm. The data were normalized to total protein concentrations, as determined using the protein assay kits (BCA protein assay; Pierce, Thermo Scientific), according to manufacturer instructions. The E1-S transport was studied as two to three independent experiments.

### Statistical Analysis

For evaluation of gene expression, RL95-2 and KLE cells were cultured independently on four different occasions; three times in duplicate, and once singly. The expression of the genes of interest and the reference genes was determined in each of these seven samples of three technical triplicates. The means for each independent experiment (n = 4) were considered in the statistical analysis. Western blotting was performed on protein samples from three biological replicates of each cell line. Cells for LC-HRMS and LC-MS/MS analyses were cultured in two and three independent experiments, respectively (n = 2 and 3), each in duplicate. E1-S uptake was performed independently two to three times, each time as triplicates.

Statistical evaluation was carried out using the GraphPad Prism software for Windows, version 8.0 (San Diego, CA, United States), with Mann–Whitney tests, Kruskal–Wallis tests followed by Dunn’s multiple comparisons tests or ANOVA followed by Tukey’s tests. Differences with *p* < 0.05 are considered as statistically significant. Unless stated otherwise, all of the data are shown as means ± standard deviation (SD).

## Results

Here, we evaluated the expression of 51 genes that encode uptake and efflux transporters of sulfated steroid precursors, estrogen biosynthetic enzymes, estrogen receptors, and phase I and II estrogen metabolic enzymes, using model cell lines of moderately and poorly differentiated EC, as RL95-2 and KLE cells, respectively. The expression of these genes was compared on the basis of the changes from the moderately differentiated to poorly differentiated EC cells, as poorly differentiated EC cells KLE *vs* the moderately differentiated EC cells RL95-2. Furthermore, each of the RL95-2 and KLE cell lines were compared directly with HIEEC cells, which provided a model cell line of normal proliferative endometrium. For these comparison purposes, the raw gene expression data for HIEEC cells were obtained from our previously published studies (Hevir-Kene and Rižner, 2015; Pavlič et al., 2021). The data for all three cell lines were here normalized to the same house-keeping gene, *HPRT1*.

### Differential Expression of E1-S Uptake Transporters in the RL95-2 and KLE Moderately and Poorly Differentiated Endometrial Cancer Cell Lines

**FIGURE 1:**
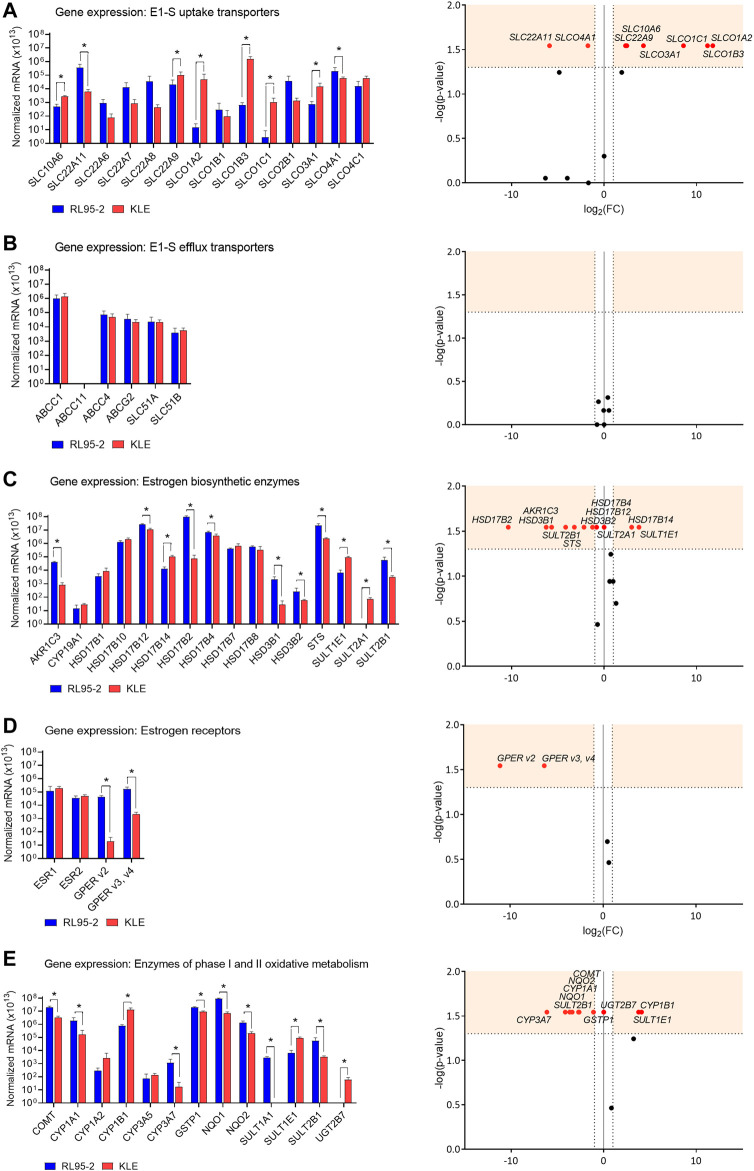
Expression of genes that encode E1-S uptake **(A)** and efflux **(B)** transporters, estrogen biosynthetic enzymes **(C)**, estrogen receptors **(D)** and enzymes of phase I and II oxidative metabolism **(E)**. Left: Expression levels in RL95-2 and KLE cells, as normalized to the three reference genes, *POLR2A, HPRT1*, and *RPLP0* (n = 4). *, *p* < 0.05 (Mann–Whitney tests). Right: Volcano plots for relative expression of the genes (KLE *vs* RL95-2 cells). FC, fold change; horizontal dashed line, cut-off for experimental significance (shaded; -log (1.3); *p* < 0.05); vertical dashed lines, cut-off for genes similarly expressed in both cell lines (FC, ±2.0); vertical line (x = 0), genes not expressed in either cell line; red symbols, differentially expressed genes; black symbols, non-differentially expressed genes.

After menopause, the ovaries cease to produce estrogens, and local E2 formation relies on the actions of transporters for the uptake of the inactive steroid precursors DHEA-S and E1-S into cells. We evaluated here the expression of 20 genes that encode 15 E1-S uptake and four E1-S efflux transporters (Figure 1, Supplementary Table S2). The great majority of the genes that encoded these E1-S uptake and efflux transporters were expressed in both the RL95-2 and KLE cells.

For the relative expression of the poorly differentiated (KLE) EC cells compared to the moderately differentiated (RL95-2) EC cells (i.e., KLE *vs* RL95-2 relative expression), differences were seen for several of the E1-S uptake transporters (Figure 1A). These showed higher expression for six E1-S uptake transporters (KLE *vs* RL95-2): *SLCO1A2* (3433.6-fold), *SLCO1B3* (2301.5-fold), *SLCO1C1* (381.2-fold), *SLCO3A1* (19.0-fold), *SLC10A6* (5.5-fold), and *SLC22A9* (5.0-fold). Two E1-S uptake transporters showed lower expression instead (KLE *vs* RL95-2): *SLC22A11* (59.3-fold) and *SLCO4A1* (3.4-fold) (Supplementary Table S2). No differences were seen for the E1-S efflux transporters (Figure 1B).

**FIGURE 2:**
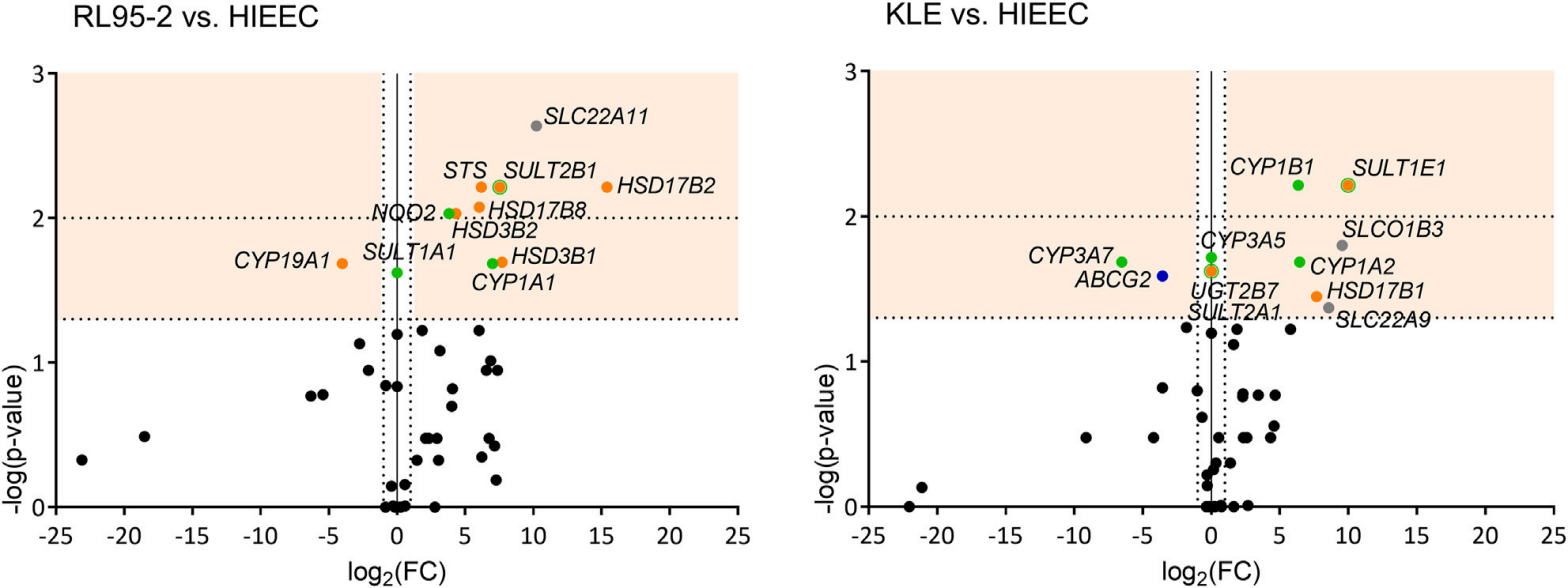
Volcano plots of genes differentially expressed in each of RL95-2 and KLE cells *vs* HIEEC cells. Genes with significant changes in expression are shown in different colors according to function: uptake of steroid precursors, gray; efflux of steroid precursors, dark blue; estradiol biosynthesis, orange; phase I and II metabolism, green. See legend to Figure 1 for further details.

Comparisons of these RL95-2 and KLE cells with the model of normal endometrium, HIEEC cells, showed several differences in the expression of the E1-S uptake transporters (Figure 2, Supplementary Table S3). For RL95-2 *vs* HIEEC cells, the relative expression was higher for *SLC22A11* (1199.8-fold). Then for KLE *vs* HIEEC, this was higher for *SLCO1B3* (754.9-fold) and *SLC22A9* (380.3-fold). For the efflux transporters, for KLE *vs* HIEEC cells, the relative expression was lower for *ABCG2* efflux transporter (11.9-fold).

This gene expression analysis thus initially indicated that both of these EC cell lines can carry out E1-S uptake. The increased relative expression of six of these E1-S uptake transporters in the poorly differentiated KLE EC cells *vs* the moderately differentiated RL95-2 EC cells, and the higher expression of two of these genes for KLE cells *vs* HIEEC cells (over RL95-2 cells *vs* HIEEC cells) imply that more sulfated steroid precursors can enter KLE cells.

### Higher Expression of STS and Negligible Expression of *CYP19A1* in RL95-2 and KLE Endometrial Cancer Cell Lines Support the Importance of the Sulfatase Pathway for E2 Formation

After the steroid precursors enter the cells, these can be further transformed through multiple steps into the most potent estrogen, E2. This can occur *via* the sulfatase and aromatase pathways, where E2 can be formed from E1-S and from DHEA-S (*via* androstenedione), respectively. The expression of 16 genes of these pathways was examined here for the RL95-2 and KLE model cell lines.

Both RL95-2 and KLE cells showed 10^5^-fold–10^6^-fold higher expression of *STS* compared to *CYP19A1* (Figure 1C). This suggests that these cell lines form estrogens only through the sulfatase pathway. Additionally, these data indicated that RL95-2 and KLE cells differ in their E2 formation. KLE *vs* RL95-2 relative expression was lower for the sulfatase pathway *STS* (9.3-fold), as also for the aromatase pathway *HSD3B1* (76.2-fold)*, HSD3B2* (4.5-fold), and *AKR1C3* (50.7-fold). For AKR1C3 lower protein levels were seen in KLE cells as compared to RL95-2 (Supplementary Figures S1A, S2).

The genes that encode the HSD17B enzymes were also differentially expressed. The greatest difference for KLE *vs* RL95-2 cells was lower relative expression for *HSD17B2* (1303.7-fold), the enzyme product of which has the highest catalytic efficiency for inactivation of E2 to E1. Similarly, this was lower for another oxidative HSD17B, *HSD17B4* (1.8-fold). On the other hand, this was higher for *HSD17B14* (7.9-fold), which also catalyzes oxidation of E2 to E1, but with lower catalytic efficiency. Additionally, KLE *vs* RL95-2 relative expression was lower for *HSD17B12* (2.4-fold), which encodes an enzyme for reduction of E1 to E2. The gene that encodes the major reductive HSD17B1 enzyme was not differentially expressed. For KLE cells, these alterations in gene expression of the reductive and oxidative HSD17B enzymes would shift the balance between E1 and E2 towards E2 formation.

Differences in KLE *vs* RL95-2 relative expression of genes for the sulfotransferase enzymes were also seen. The relative expression here was lower for the gene that encodes DHEA sulfotransferases, *SULT2B1* (17.7-fold), while expression of *SULT2A1* was only detected in KLE cells. Higher relative expression was instead seen for the gene encoding E1 and E2 sulfotransferase, *SULT1E1* (13.6-fold). This differential expression of these genes suggests that KLE cells followed more sulfation of DHEA (higher relative expression of *SULT2A1*), E1 and E2 (higher relative expression of *SULT1E1*) compared to RL95-2 cells.

Comparing RL95-2 cells to HIEEC cells, relative expression was lower for *CYP19A1* (16.1-fold), and higher for *STS* (73.1-fold) (Figure 2, Supplementary Table S3). This suggests that for RL95-2 cells, E1 and E2 can be formed *via* the sulfatase pathway. Several other genes involved in the aromatase and the sulfatase pathways also showed higher relative expression here: *HSD17B2* (43,060.2-fold)*, HSD3B1* (209.0-fold), and *SULT2B1* (185.8-fold). Other genes with higher relative expression in RL95-2 cells *vs* HIEEC cells were *HSD17B8* (65.4-fold) and *HSD3B2* (19.9-fold). This higher expression of *HSD3B1* and *HSD3B2* suggests that in RL95-2 cells, DHEA can be metabolized to androstenedione, although DHEA can also be sulfated due to higher expression of *SULT2B1*. Higher expression of several genes for oxidative HSD17B enzymes suggest that for RL95-2 cells compared to HIEEC cells, the balance between E1 and E2 will be shifted towards E1.

For KLE *vs* HIEEC cells, relative expression was higher for *HSD17B1* (204.6-fold), which encodes reductive HSD17B, *SULT1E1* (1020.7-fold), and *SULT2A1*, which was expressed in KLE cells but not in HIEEC cells. These data indicate that for KLE cells, more E2 can be formed, but conjugation to E2-S would prevail.

### RL95-2 and KLE Cell Line Models of Moderately and Poorly Differentiated Endometrial Cancer Express *ESR1* and *ESR2*, but Differ in *GPER* Expression

The actions of estrogens are mediated through their binding to and activation of nuclear estrogen receptors and membrane-bound receptors. Here, expression was evaluated for *ESR1* and *ESR2*, which encode nuclear receptors ERα and ERβ, respectively, and the three *GPER* transcript variants, as *v2* and *v3* plus *v4* (Hevir-Kene and Rižner, 2015) (Figure 1D).

Both *ESR1* and *ESR2* were expressed in RL95-2 and KLE cells, with no difference between these. For KLE *vs* RL95-2 relative expression, this was lower for *GPER v2* (2189.7-fold) and *GPER v3* plus *v4* (80.9-fold). When RL95-2 and KLE cells were compared to the control cell line, HIEEC cells, there were no significant differences in gene expression of estrogen receptors *ESR1*, *ESR2* and *GPER* (Figure 2, Supplementary Table S3). The expression of *ESR1* surmounted expression of *ESR2* in all three cell lines RL95-2, KLE and HIEEC for 3-fold, 3-fold and 67-fold, respectively (Supplementary Table S4).

These data suggest that in moderately differentiated EC (RL95-2 cells), estrogens might act *via* nuclear and membrane bound receptors, while in poorly differentiated EC (KLE cells), estrogens might preferentially activate the nuclear receptors.

### Decreased Expression of Genes Encoding Phase II Enzymes in the KLE Cell Line, as a Model of Poorly Differentiated Endometrial Cancer

For phase I metabolism inside cells estrogens can be metabolized to 2-, 4- or 16α-hydroxy-estrogens by the actions of different CYP enzymes. Catechol estrogens (2- or 4-OH-E1/E2) can be oxidized to semiquinones or quinones, which are associated with DNA damage. To avoid cell damage, these catechols and quinones are further conjugated and detoxified in phase II metabolism, by the COMT, SULT, UGT, GSTP1, NQO1, and NQO2 enzymes (Rižner, 2013; Hevir-Kene and Rižner, 2015). The expression of 13 genes that encode phase I and II metabolic enzymes was evaluated in these model cell lines (Figure 1E).

The relative expression of genes involved in phase I metabolism for KLE *vs* RL95-2 cells was higher for *CYP1B1* (17.1-fold), which encodes a 4-hydoxylase, but lower for *CYP1A1* (10.6-fold) and *CYP3A7* (70.1-fold)*,* which encode a 2-hydroxylase and a 16α-hydroxylase, respectively.

For expression of the eight genes of phase II metabolism that were evaluated, KLE *vs* RL95-2 relative expression was higher for *SULT1E1* (13.6-fold), while expression of *UGT2B7* was only detected in KLE cells. All of the other genes that encode detoxification-associated enzymes showed lower relative expression for KLE *vs* RL95-2 cells, as *SULT2B1* (17.7-fold), *NQO1* (12.7-fold), *NQO2* (6.6-fold), *COMT* (6.3-fold), and *GSTP1* (2.2-fold), with *SULT1A1* not expressed in KLE cells. This was confirmed at the protein level for soluble COMT in KLE as compared to RL95-2 cells (Supplementary Figures S1B, S3). These changes imply that more 4-hydroxy-estrogens can be formed in KLE cells, along with more genotoxic 3,4-quinones, as a result of lower expression of genes that encode phase II metabolic enzymes.

Comparisons of RL95-2 and KLE cells with HIEEC cells for expression of genes encoding phase I metabolic enzymes revealed some significant differences (Figure 2, Supplementary Table S3). RL95-2 *vs* HIEEC relative expression was higher for *CYP1A1* (127.6-fold), while for KLE *vs* HIEEC cells, this was higher for *CYP1A2* (87.2-fold), *CYP1B1* (81.1-fold) and *CYP3A5* which was expressed in KLE but not in HIEEC cells. On the other hand, KLE cells showed lower relative expression for *CYP3A7* (93.2-fold). These data suggest that more 2-hydroxyestrogens can be formed in RL95-2 *vs* HIEEC cells, and more 4-hydroxyestrogens in KLE *vs* HIEEC cells.

Genes of phase II metabolism were also differentially expressed between each of these EC cell lines and HIEEC cells (Figure 2, Supplementary Table S3). RL95-2 cells showed higher relative expression of *SULT2B1* (185.8-fold) and *NQO2* (14.1-fold), as for KLE cells for *SULT1E1* (1020.7-fold) and *UGT2B7* which was expressed in KLE cells but not in HIEEC cells.

### Gene Expression Analysis Indicates Increased E1-S Uptake, Alteration in E2 Biosynthesis and Metabolism, and Decreased Detoxification of Estrogen Quinones in KLE Cells Compared to RL95-2 Cells

**FIGURE 3:**
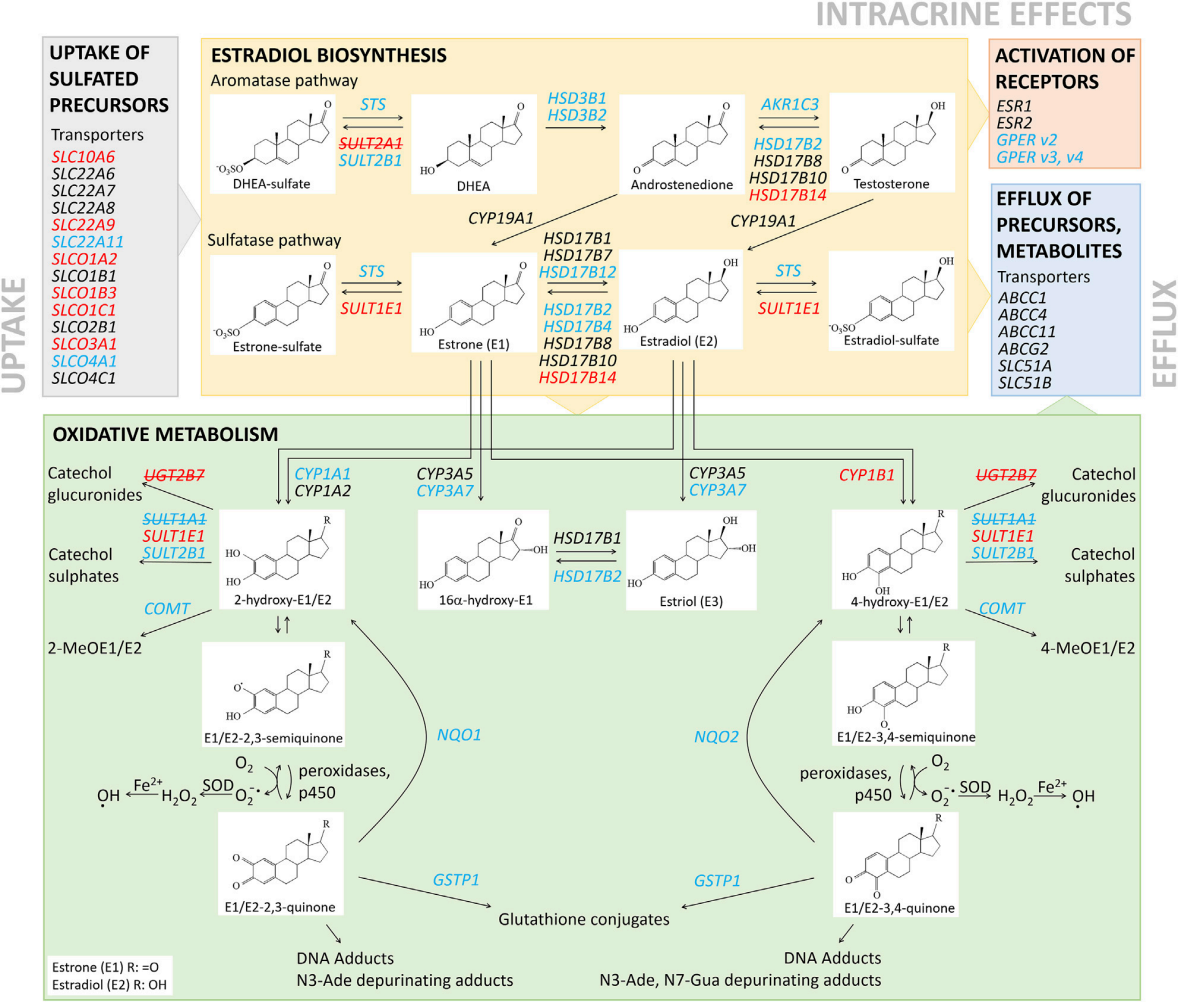
Schematic representation of local estradiol biosynthesis and metabolism in RL95-2 and KLE cells. Genes with higher and lower relative expression for KLE *vs* RL95-2 cells are indicated in red and blue, respectively. Genes in red and blue with strikethrough were not expressed in either in RL95-2 or KLE cells, respectively.

**FIGURE 4:**
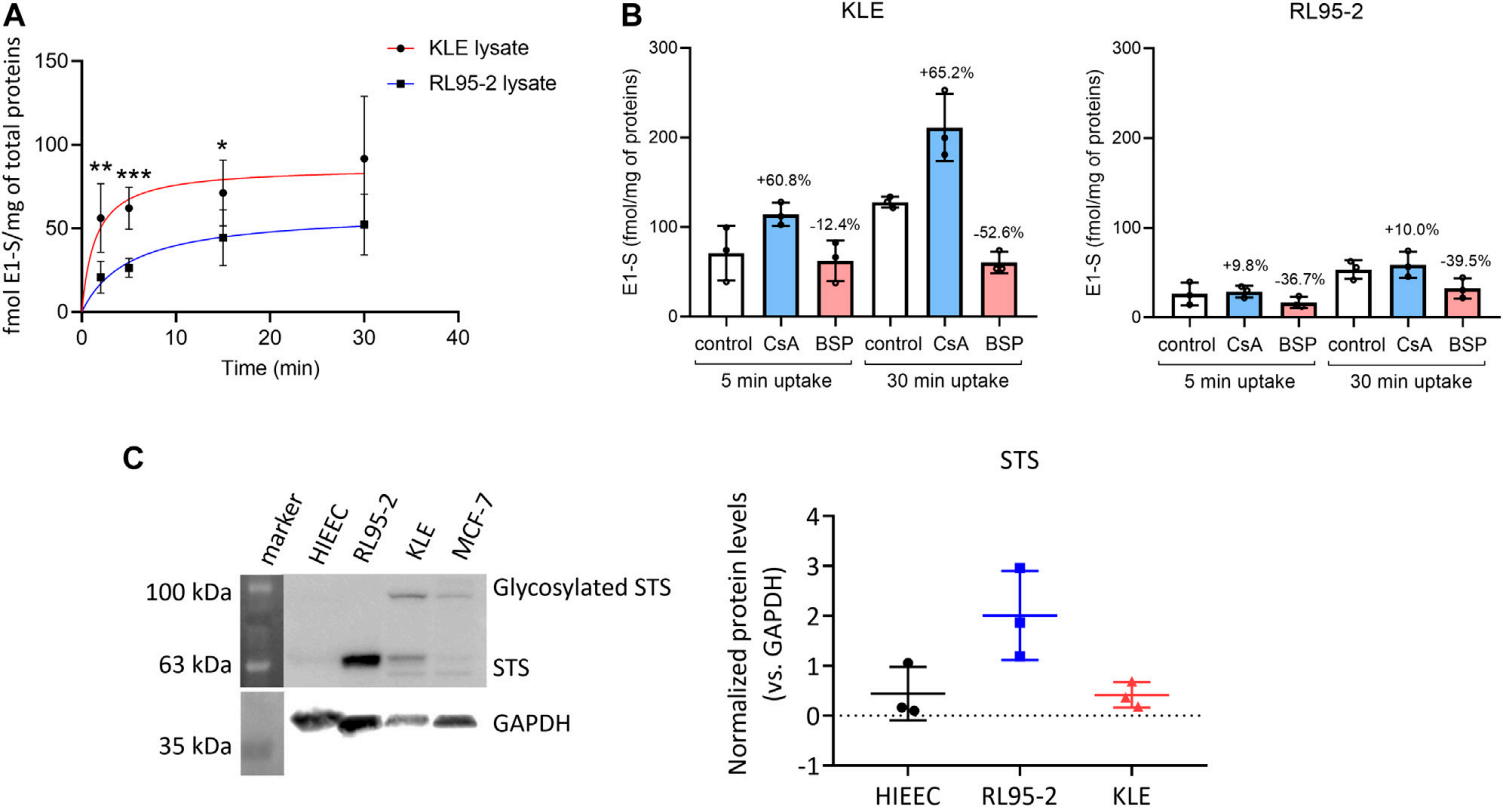
**(A)** Time-course of [^3^H]E1-S uptake in RL95-2 and KLE cells at 37°C (as total transport). Data are means ± SD, for two to three independent experiments, with each carried out in triplicate. Nonlinear regression curve fitting of the data is also shown (GraphPad Prism for Windows, version 8.4.3; San Diego, CA, United States). **, *p* ≤ 0.01 (Mann–Whitney U tests). **(B)** E1-S uptake in the presence of OATP inhibitors 10 µM cyclosporine A (CsA) and 10 µM bromosulphophthalein (BSP) in KLE **(left)** or RL95-2 **(right)** cell lines at two different time points, 5 and 30 min. Control cells were treated with 16 nM [^3^H]E1-S without inhibitor. Percentages were calculated based on E1-S uptake in control at the evaluated time point. Statistical analysis was done using Mann Whitney U tests. Results of three individual experiments in two technical replicates. **(C)** Protein levels of sulfatase (STS) in HIEEC, RL95-2, and KLE cells. Left: Representative Western blot. Right: Quantification of STS levels, normalized to GAPDH as control, with three biological replicates for each cell line (Kruskal–Wallis tests, with Dunn’s multiple comparisons). For whole Western blots and densitometry readings see Supplementary Figure S5.

The changes in the expression of genes that encode uptake and efflux transporters, estradiol biosynthetic enzymes, and phase I and II metabolic enzymes (Figure 1; summarized in Figure 3) were then integrated and taken forward. These indicated that for KLE *vs* RL95-2 cells, the KLE cells would initially have increased uptake of E1-S (i.e., higher relative expression of *SLC10A6, SLC22A9, SLCO1C1, SLCO1A2, SLCO3A1, SLCO1B3*) and decreased formation of E1 (i.e., lower relative expression of *STS* and lower protein levels of STS (Figure 4C). This can then be combined with increased activation of E1 to E2 (i.e., lower relative expression of *HSD17B2*) and increased formation of sulfated estrogens (i.e., higher relative expression of *SULT1E1* and nonsignificantly higher protein levels (Figure 1, Supplementary Figures S1, S4). Indeed, we also experimentally confirmed increased E1-S uptake in KLE *vs* RL95-2 cells here (Figures 4A,B), which is associated with higher expression of several genes encoding OATP transporters, especially *SLCO1A2*, *SLCO1B3* and *SLCO1C1* in KLE, as also confirmed by inhibition of E1-S uptake by bromosulphophthalein, a known inhibitor of OATP1B1, OATP1B3, OATP1A2, OATP2B1 (König et al., 2006). Increased E1-S uptake in KLE cells in the presence of cyclosporine A, which also inhibits E1-S efflux transporters (Dantzic et al., 2018), imply that these efflux ABC transporters importantly regulate E1-S uptake in KLE cells. Furthermore, for KLE cells, the active estrogens formed from E1-S *via* the sulfatase pathway would be expected to act *via* nuclear receptors ERα and ERβ (i.e., lower relative expression of *GPER*, compared with RL95-2 cells).

Additionally, again for KLE *vs* RL95-2 cells expression data suggest that the KLE cells may show lower levels of 2-hydroxyestrogens and 16α-hydroxyestrogens (i.e., lower relative expression of *CYP1A1*, *CYP3A7*), but more 4-hydroxycatechols (i.e., higher relative expression of *CYP1B1*), which can be oxidized to carcinogenic 3,4-quinones. In KLE cells, lower detoxification levels of catechols (i.e., lower relative expression of *COMT* and lower S-COMT protein levels, Supplementary Figure S1) and quinones (i.e., lower relative expression of *NQO1, NQO2, GSTP1*) would also indicate higher levels of DNA adducts, compared to RL95-2 cells.

A similar summarized comparison between RL95-2 and HIEEC cells (Figure 2, Supplementary Figure S6) suggests enhanced hydrolysis of E1-S to E1 (i.e., increased levels of STS), hindered reduction of E1-S to E2 (i.e., higher expression of *HSD17B2*) and hydroxylation of E1 at the C2 position to form 2-hydroxyestrogens (i.e., higher expression of *CYP1A1*).

For the comparison between KLE and HIEEC cells (Figure 2, Supplementary Figure S7), this would support increased E1-S uptake (i.e., high relative expression of *SLCO1B3, SLC22A9*), but also enhanced activation of E1 to E2 and conjugation to E2-S, or formation of 2-hydroxy or 4-hydroxy-estrogens (i.e., higher relative expression of *HSD17B1, SULT1E1, CYP1A2*, *CYP1B1*).

### In RL95-2 and KLE Cells as Model Cell Lines of Moderately and Poorly Differentiated Endometrial Cancer, Estrogens Are Not Formed From DHEA-S and DHEA

**FIGURE 5:**
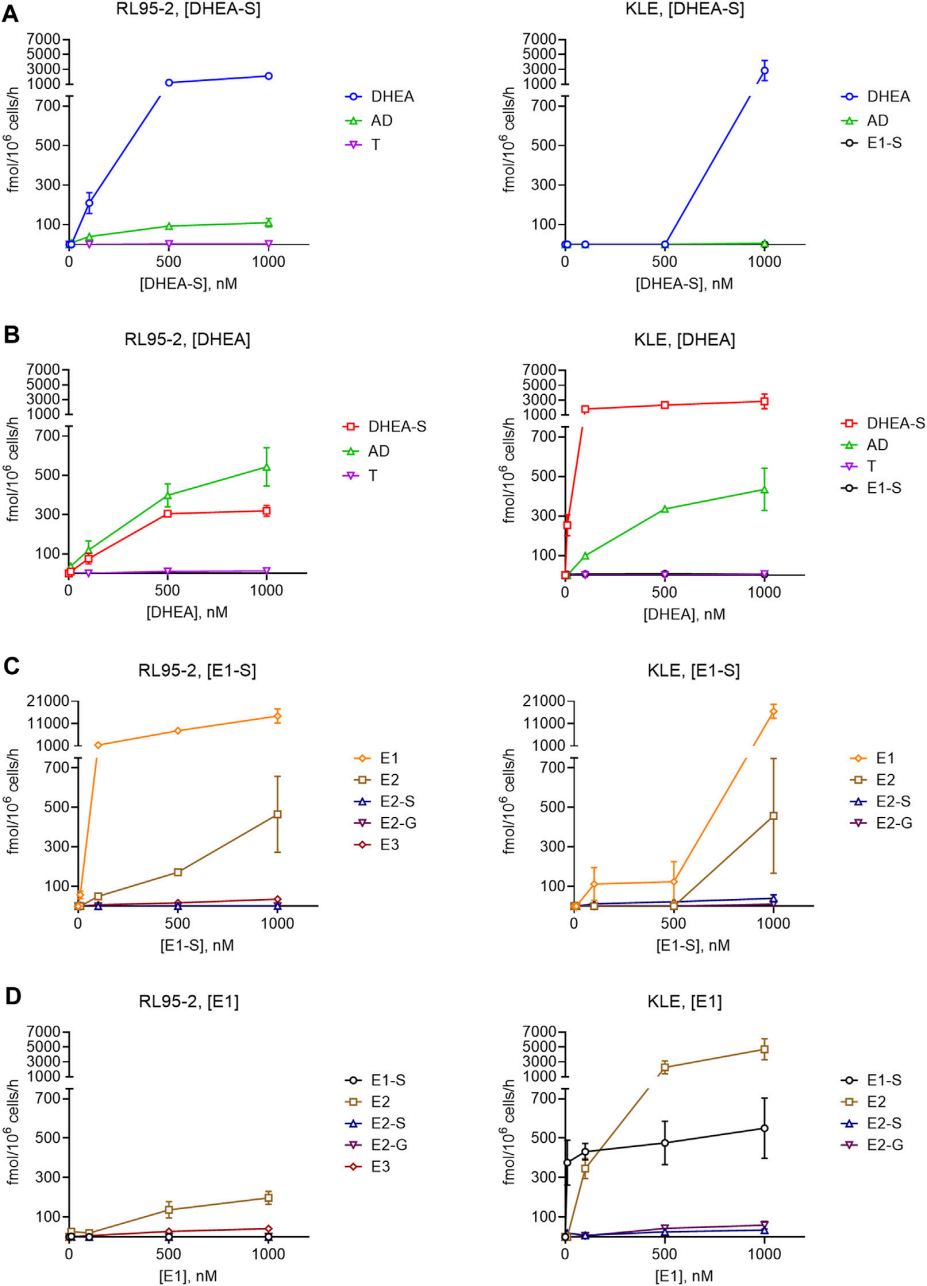
Metabolism of DHEA-S **(A)**, DHEA **(B)**, E1-S **(C)** and E1 **(D)** in RL95-2 **(left)** and KLE **(right)** cells. Metabolites were separated and quantified by LC-HRMS. AD, androstenedione; E2-G, E2-glucuronide; E3, estriol; T, testosterone; for other abbreviations see main text.

To evaluate the formation of active estrogens in the RL95-2 and KLE cells, the metabolism of 10 nM, 100 nM, 500 nM, and 1000 nM DHEA-S, DHEA, E1-S, and E1 was studied following their addition to the cells, with their products separated and quantified by LC-HRMS (Figure 5).

These data showed more efficient DHEA-S metabolism in RL95-2 cells compared to KLE cells. In RL95-2 cells, DHEA-S was metabolized to DHEA and lower levels of androstenedione. In KLE cells, DHEA-S was only metabolized to DHEA at the highest, non-physiological, DHEA-S concentration (i.e., 1,000 nM), which is in line with the lower levels of STS seen previously for KLE cells (Figure 4)*.*

In contrast, metabolism of DHEA was more efficient in KLE cells, where DHEA-S was the major product, followed by androstenedione. Here, DHEA-S production can be explained by expression of *SULT2A1*. In RL95-2 cells, DHEA was instead mainly metabolized to androstenedione, with very low levels of testosterone. Here, in RL95-2 cells, the lower DHEA-S production is supported by the higher relative expression of *HSD3B1, HSD3B2*, and *AKR1C3*. These data confirm that DHEA-S and DHEA cannot serve as precursors for estrogen biosynthesis in these cells, as no estrogens were formed here from these steroid precursors.

### In RL95-2 and KLE Cells as Model Cell Lines of Moderately and Poorly Differentiated Endometrial Cancer, E1-S Is Metabolized to Active Estrogens

Incubations with E1-S revealed that both EC cell lines can take up and metabolize the E1-S steroid precursor, to form active E2. For KLE *vs* RL95-2 cells, lower levels of E1 and E2 were seen, with E2 formed only at the supraphysiological 1000 nM E1-S. This can be explained by higher relative expression of STS in RL95-2 cells. In contrast, incubation of EC cell lines with E1 showed that in KLE cells, there is greater metabolism of E1 to E2 and E1-S, which would be due to lower expression of *HSD17B2* and higher expression of *SULT1E1*, compared to RL95-2 cells. In RL95-2 cells, formation of very low levels of estriol was seen after addition of E1-S and E1, which is in line with higher relative expression of *CYP3A7*, and for KLE cells, E2-glucuronides were detected as a result of UGT2B7 activity (Figure 1).

**FIGURE 6:**
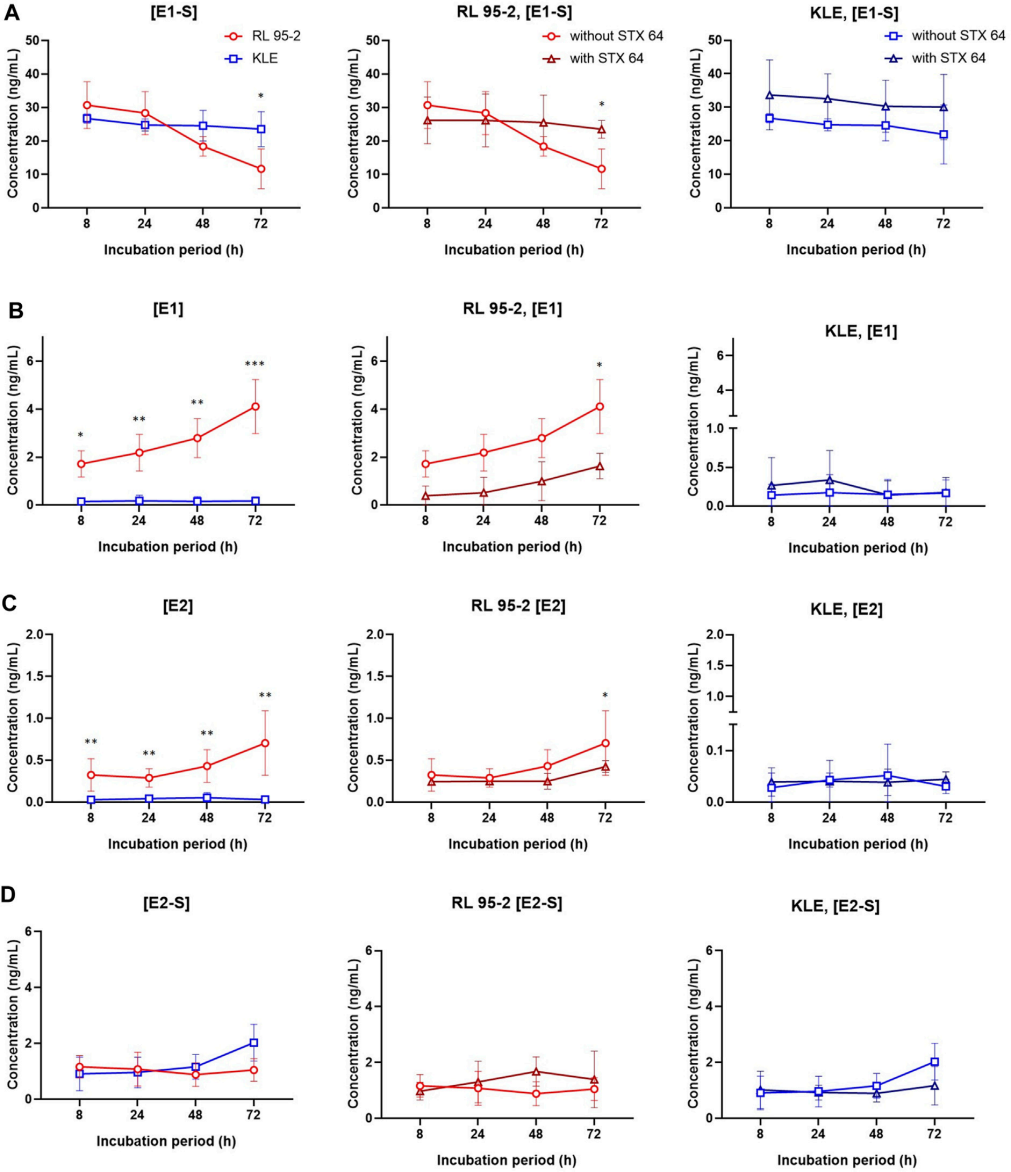
E1-S metabolism in RL95-2 and KLE cells. Time courses for the estrogen metabolites following addition of 2.3 nM E1-S to the cells, for E1-S **(A)**, E1 **(B)**, E2 **(C)**, and E2-S **(D)**, in the presence and absence of 10 nM STX64 (sulfatase inhibitor). Data are means ± SD. *, *p* < 0.05; **, *p* < 0.01; ***, *p* < 0.001 (ANOVA plus Tukey’s tests).

To better simulate the physiological conditions, the metabolism of E1-S in these EC cell lines was also studied at lower E1-S concentrations: 2.3, 8.5, and 85 nM (Figure 6, Supplementary Figure S8). After 72 h for KLE *vs* RL95-2 cells, metabolism of 2.3 nM E1-S resulted in lower levels of E1, E2 and E2-S; for KLE cells here, E1 and E2 were formed, but E2-S was the major product. The profiles of the metabolites remained the same at the higher, 8.5 and 85 nm E1-S concentrations (Supplementary Figure S8).

When comparing the EC cell lines to HIEEC cells, more E1 and E2 were formed from E1-S for RL95-2 cells, which is in line with higher levels of STS, and more E2-S was formed for KLE cells, which can be explained by higher relative expression of *HSD17B1* and *SULT1E1* (Figure 2, Supplementary Figures S6, S7). The importance of the sulfatase pathway for formation of E2 from E1-S was additionally confirmed using a specific and potent STS inhibitor, STX64; this shows an IC_50_ of 8 nM at 20 μM E1-S (Malini et al., 2000), and was used at 10 nM STX64. In RL95-2 cells, this addition of STX64 in combination with 2.3 nM E1-S resulted in an almost complete block of the sulfatase pathway, with significantly lower levels of E1 and E2 formed (Figure 6). In KLE cells, where less E1 was formed from E1-S, addition of STX64 affected the levels of E1 and E2, although a significant difference was only seen for E1-S (Figure 6).

## Discussion

The current understanding of estrogen formation and actions in moderately and poorly differentiated EC is very limited (Berstein et al., 2003). We aimed to contribute to the clarification of estrogen formation from steroid precursors DHEA-S, DHEA, E1, and E1-S in the RL95-2 and KLE cell lines, as models of moderately differentiated and poorly differentiated metastatic (Van Nyen et al., 2018) EC, respectively. We focused on two well-characterized cell lines that are commonly used, uncontaminated, with known STR profile and histopathological characteristics. Although with these two model cell lines we did not cover inter-individual variability among patients, the results of our study may help to determine whether blockade of estrogen actions at the pre-receptor or receptor levels can be considered as new options for treatments of individual patients with these histological types of EC.

Transport of steroid sulfates *via* organic anion transporters (*SLC*), organic anion transporter polypeptides (*SLCO*), ABC transporters, and OSTαβ (dimer *SLC15A*, *SLC51B*) is an important process after menopause, which is when production of lipid-soluble estrogens diminishes, and the water-soluble sulfated precursors, DHEA-S and E1-S, are still present at relatively high levels in the plasma (Rižner et al., 2017). In the present study, we evaluated the expression of 19 transporters encoded by 20 genes.

Comparisons between these EC cell lines suggested increased uptake of E1-S and DHEA-S in the poorly differentiated KLE EC cells, as six uptake transporters (i.e., *SLCO1A2, SLCO1B3, SLCO1C1, SLCO3A1, SLC10A6*, *SLC22A9*) showed higher relative expression in KLE cells *vs* the moderately differentiated RL95-2 EC cell line, and as the expression of the efflux transporters was not different. These expression data were also supported by E1-S uptake inhibition by bromosulphophthalein and by the E1-S uptake studies that show increased influx in KLE cells compared to RL95-2 cells. Comparisons of gene expression in these EC cell lines with that of the control HIEEC cell line indicated their increased uptake of steroid precursors. This was due to higher expression of *SLC22A11* in RL95-2 cells and *SLCO1B3* and *SLC22A9* in KLE cells, *vs* HIEEC cells. This would be combined with decreased efflux of the steroid precursors in the EC cells, as the gene for efflux transporter *ABCG2* showed lower expression in KLE cells, and the gene for *ABCC11* was not expressed in RL95-2 and KLE cells. All in all, these gene expression and functional studies confirm E1-S uptake in these moderately and poorly differentiated EC cells, with higher uptake seen in the poorly differentiated KLE EC cells.

These differences in expression of the transporters might also be associated with menopausal status of the patients who donated tissues for the original establishment of these HIEEC, RL95-2, and KLE cell lines. The HIEEC cell line was derived from the proliferative endometrium of a 37-year-old woman (Chapdelaine et al., 2006), and the RL95-2 and KLE cell lines were established from tissues of postmenopausal patients. However, to the best of our knowledge, there are no suitable control endometrial cell lines that would originate from postmenopausal women. We recently investigated the HEC-1A cell line, a model of postmenopausal well-differentiated EC cells. Compared with HIEEC cells, HEC-1A cells showed higher expression of *SLCO1B3*, followed by *SLCO1B1* and *SLCO2B1,* where silencing of *SLCO1B3* decreased E1-S uptake. Comparison of Ishikawa cells, which are a model for premenopausal EC, *vs* HIEEC cells as the control again, showed higher expression for *SLCO1C1* and *SLCO1A2*, but lower expression of *SLCO3A1*. *ABCG2*, which encodes an efflux transporter, showed lower expression in both HEC-1A and Ishikawa cells, compared to HIEEC cells (Pavlič et al., 2021). These data support the role of these SLCO and ABC transporters in EC.

We and others have previously reported on the importance of the sulfatase pathway for E2 formation in EC tissue samples and model cell lines (Hevir-Kene and Rižner, 2015; Sinreih et al., 2017; Cornel et al., 2018). The crucial role of sulfatase was also confirmed in mouse endometrial cancer xenograft model where STS inhibitor STX64 significantly inhibited tumour growth (Foster et al., 2008). STS inhibitor STX64, also known as irosustat, has also been investigated in a phase II clinical study (NCT00910091) in patients with advanced/metastatic or recurrent endometrial cancer. In this study irosustat showed lower clinical benefit compared to progestin megestrol acetate. However, expression of STS has not been determined in these patients, which can explain the observed weak response to irosustat. Similarly as shown here for KLE, patients with metastatic cancer probably had low levels of STS in cancer tissue, and varied STS levels in other tissues.

Here we confirmed the crucial role of the sulfatase pathway once again, with 10^5^-fold–10^6^-fold higher expression of *STS* compared to *CYP19A1* in both RL95-2 and KLE cells. Gene expression analysis indicated that E2 can be formed from E1-S in both of these EC cell lines. In KLE *vs* RL95-2 cells, lower levels of E1 would be expected due to lower relative expression of *STS*, but higher activation to E2 would be expected due to lower relative expression of the major oxidative *HDS17B2* and *HSD17B4* genes (Rižner, 2013). Additionally, when compared to HIEEC cells, KLE cells showed higher expression of the major reductive *HSD17B1* gene, which supports higher E2 formation, and higher expression of *SULT1E1*, which leads to E2-S as the major product of E1 metabolism and questions the action of estrogens in this cell line. The E1-S metabolism supported these data, with production of E1, E2, and very low levels of estriol in RL95-2 cells, and production of E1, E2 and E2-S in KLE cells. The metabolism of E1 was more efficient in KLE cells, with the formation of E2, E1-S, E2-S, and E2-glucuronide, while in RL95-2 cells, E2, estriol, and E1-S were formed. At 2.3 nM E1-S, E1 and E2 were the main products for RL95-2 cells, and E2-S for KLE cells. For RL95-2 cells *vs* HIEEC cells, there was >85-fold higher expression of *STS*, with increased STS protein levels also seen. This indicates enhanced hydrolysis of E1-S to E1 in RL95-2 cells, and supports the importance of the sulfatase pathway. This was also confirmed by the E1-S metabolism in the presence of the STS inhibitor STX64. For KLE *vs* HIEEC cells, there were no significant differences in expression of *STS,* while there was higher expression of *HSD17B1* and *SULT1E1*, thus allowing hydrolysis of E1-S to E1, and promoting formation of E2 and E2-S. These data confirm that these cell lines of moderately and poorly differentiated EC have a capacity for E1-S metabolism and E2 formation.

Also, higher expression of DHEA sulfotransferases supported the biosynthesis of E2 from E1-S *via* the sulfatase pathway. *SULT2B1* was expressed in both RL95-2 and KLE EC cell lines, with higher relative expression in RL95-2 cells *vs* KLE and HIEEC cells, while the more efficient *SULT2A1* (Lu et al., 2008) was expressed only in KLE cells. These data suggest that in KLE cells, DHEA sulfation would prevail over hydrolysis of DHEA-S. In RL95-2 *vs* HIEEC cells, higher expression of *HSD3B1* and *HSD3B2* with concurrent higher expression of *HSD17B2* and *HSD17B8* imply that DHEA metabolism proceeds to androstenedione, while lower relative expression of *AKR1C3* in KLE *vs* RL95-2 cells suggests that more testosterone will be formed in the moderately differentiated RL95-2 cells. This supports the protective role of androgens in EC (Gibson et al., 2014; Simitsidellis et al., 2017). The results of the DHEA-S and DHEA metabolism studies are also in line with the gene expression data. In RL95-2 cells, the balance between hydrolysis of DHEA-S and sulfation of DHEA was shifted towards hydrolysis to DHEA, with further metabolism to androstenedione and testosterone; instead, in KLE cells, sulfation prevails.

Binding of estrogens to their receptors is crucial for estrogen actions. In our previous study, *ESR1* and *ESR2* showed higher expression in Ishikawa *vs* HIEEC cells, while *ESR1* was not expressed in HEC-1A cells (Hevir-Kene and Rižner, 2015). Here, both the RL95-2 and KLE moderately and poorly differentiated EC cell lines expressed the genes that encode ERα and ERβ and the expression of *ESR1* prevailed. For these RL95-2 and KLE EC cell lines compared to the model HIEEC cell line, there was no difference in expression of *ESR1*. This is surprising, as differences in ERα have previously been associated with histology, response to therapy, and metastatic potential of EC (Swasti, 2018). Previously, we showed lower *GPER* expression in HEC-1A *vs* HIEEC cells (*v2*), and *vs* Ishikawa cells (*v3*, *v4*) (Hevir-Kene and Rižner, 2015). For Ishikawa and HEC-1A cells, other studies have reported that E2 and 4-hydroxytamoxifen stimulate cell proliferation *via* GPER and *via* the MAPK and PI3K pathways (Vivacqua et al., 2006). In the present study, there were important difference in *GPER* expression, with lower relative expression for KLE *vs* RL95-2 cells, which suggests that estrogen action *via* GPER might be hampered in poorly differentiated EC. These data thus suggest that in moderately differentiated EC, estrogens might act *via* ERα and ERβ or GPER, while in poorly differentiated EC, estrogens might act preferentially *via* ERα and ERβ, and less *via* GPER. However, further studies are needed to clarify estrogen action in these cell lines.

Also phase I and phase II metabolism may differ between these EC cell lines. Compared to the KLE cells, RL95-2 cells differentially expressed genes of phase I and II metabolism in favor of 2-MeOE1/E2 formation (higher relative expression of *CYP1A1*, *COMT*) and 4-MeOE1/E2 formation (higher relative expression of *NQO2*, *COMT*). Formation of these metabolites has a protective role since harmful hydroxyl-E1/E2 are deactivated and also 2-MeOE2 is known to have antiproliferative, antiangiogenic, and proapoptotic effects (Lépine et al., 2010; Hevir et al., 2011). Expression patterns of these phase I and II metabolism genes might to some extent be associated with EC; however, on the other hand, correlations have also been reported between age of patients and formation of 2-MeO and 4-MeO E1/E2 (Brinton et al., 2016), with more 2-MeO and 4-MeO E1/E2 formed in older patients.

For KLE *vs* RL95-2 cells, lower relative expression of *CYP1A1* and *CYP3A7* and higher relative expression of *CYP1B1* are in favor of the formation of 4-hydroxyestrogens in KLE cells and 2-OH or 16αOH estrogens in RL95-2 cells. Also, KLE cells will probably form more catechol glucuronides and less 2- or 4-MeO E1/E2 and glutathione conjugates, compared to RL95-2 cells. This will be due to higher relative expression of *UGT2B7* (i.e., for catechol glucuronides) and lower relative expression of *COMT* and *GSTP1* (i.e., for 2- and 4-MeO E1/E2 and glutathione conjugates). Additionally, for KLE *vs* RL95-2 cells, lower relative expression of *GSTP1, NQO1*, and *NQO2* favors higher DNA adduct formation in poorly differentiated EC.

For KLE cells *vs* control HIEEC cells, higher expression of *CYP1A2* and *CYP1B1*, and lower expression of *CYP3A7* indicate that in KLE cells, E1 or E2 are primarily transformed into 2-OH E1/E2 or 4-OH E1/E2, and less so into 16α-OH E1/E2. Higher expression of *SULT1E1* for KLE *vs* HIEEC cells indicates increased formation of catechol sulfates, which have potential protective roles. Higher expression of *CYP1A2* and *CYP1B1* might lead to increased formation of E1/E2-2,3- or E1/E2-3,4-quinones, and thus to higher probability of the formation of depurinating estrogen–DNA adducts, which are associated with carcinogenesis (Cavalieri and Rogan, 2016). In model EC cell lines of lower grade EC, as RL95-2 (G2), HEC-1-A (G2), and Ishikawa (G1) cells (Hevir-Kene and Rižner, 2015), increased *CYP1A2* and *CYP1B2* expression has not been reported previously, compared to HIEEC cells. To clarify the formation and action of the oxidative metabolites of estrogens and their conjugates further studies are needed.

## Conclusion

Here, we carried out gene expression analysis supported by E1-S uptake, metabolism studies for DHEA-S, DHEA, E1-S, and E1, and quantification of metabolites by LC-HRMS and LC-MS/MS. These analyses have revealed that the RL95-2 and KLE model cell lines of moderately and poorly differentiated EC, respectively, differ significantly. In both of these cell lines, DHEA-S and DHEA cannot serve as precursors for estrogen formation. RL95-2 cells show metabolism of DHEA-S to DHEA and androstenedione, while KLE cells show little DHEA-S metabolism to DHEA, and DHEA-S production from DHEA, along with androstenedione. In contrast E1-S is metabolized to active estrogens in both of the RL95-2 and KLE cell lines. For RL95-2 cells, as a model of moderately differentiated EC, E1 and E2 are formed, as also for KLE cells, as a model of poorly differentiated EC; however, in KLE cells, E2-S is the major product at physiological E1-S concentrations. Lack of understanding of estrogen action in these model cell lines of moderately and poorly differentiated EC calls for further studies that may reveal new avenues for treatment.

## Data Availability

The original contributions presented in the study are included in the article/Supplementary Material, further inquiries can be directed to the corresponding author.

## References

[B1] Amant F. Moerman P. Neven P. Timmerman D. Van Limbergen E. Vergote I. (2005). Endometrial Cancer. Lancet 366 (9484), 491–505. 10.1016/s0140-6736(05)67063-8 16084259

[B2] Berstein L. M. Tchernobrovkina A. E. Gamajunova V. B. Kovalevskij A. J. Vasilyev D. A. Chepik O. F. (2003). Tumor Estrogen Content and Clinico-Morphological and Endocrine Features of Endometrial Cancer. J. Cancer Res. Clin. Oncol. 129 (4), 245–249. 10.1007/s00432-003-0427-9 12695909 PMC12161952

[B3] Bray F. Ferlay J. Soerjomataram I. Siegel R. L. Torre L. A. Jemal A. (2018). Global Cancer Statistics 2018: GLOBOCAN Estimates of Incidence and Mortality Worldwide for 36 Cancers in 185 Countries. CA: A Cancer J. Clin. 68 (6), 394–424. 10.3322/caac.21492 30207593

[B4] Brinton L. A. Trabert B. Anderson G. L. Falk R. T. Felix A. S. Fuhrman B. J. (2016). Serum Estrogens and Estrogen Metabolites and Endometrial Cancer Risk Among Postmenopausal Women. Cancer Epidemiol. Biomarkers Prev. 25 (7), 1081–1089. 10.1158/1055-9965.epi-16-0225 27197275 PMC4930692

[B5] Bustin S. A. Benes V. Garson J. A. Hellemans J. Huggett J. Kubista M. (2009). The MIQE Guidelines: Minimum Information for Publication of Quantitative Real-Time PCR Experiments. Clin. Chem. 55 (4), 611–622. 10.1373/clinchem.2008.112797 19246619

[B6] Cavalieri E. L. Rogan E. G. (2011). Unbalanced Metabolism of Endogenous Estrogens in the Etiology and Prevention of Human Cancer. J. Steroid Biochem. Mol. Biol. 125 (3-5), 169–180. 10.1016/j.jsbmb.2011.03.008 21397019 PMC4423478

[B7] Cavalieri E. L. Rogan E. G. (2016). Depurinating estrogen‐DNA Adducts, Generators of Cancer Initiation: Their Minimization Leads to Cancer Prevention. Clin. Transl. Med. 5, 12. 10.1186/s40169-016-0088-3 26979321 PMC4792821

[B8] Chapdelaine P. Kang J. Boucher-Kovalik S. Caron N. Tremblay J. P. Fortier M. A. (2006). Decidualization and Maintenance of a Functional Prostaglandin System in Human Endometrial Cell Lines Following Transformation with SV40 Large T Antigen. Mol. Hum. Reprod. 12 (5), 309–319. 10.1093/molehr/gal034 16556676

[B9] Chiang S. Soslow R. A. (2014). Updates in Diagnostic Immunohistochemistry in Endometrial Carcinoma. Semin. Diagn. Pathol. 31 (3), 205–215. 10.1053/j.semdp.2014.03.002 24951284

[B10] Cornel K. M. C. Delvoux B. Saya T. Xanthoulea S. Konings G. F. J. Kruitwagen R. P. F. M. (2018). The Sulfatase Pathway as Estrogen Supply in Endometrial Cancer. Steroids 139, 45–52. 10.1016/j.steroids.2018.09.002 30217785

[B11] Dantzic D. Noel P. Merien F. Liu D.-X. Lu J. Han H. (2018). The Effects of Synthetically Modified Natural Compounds on ABC Transporters. Pharmaceutics 10 (3), 127. 10.3390/pharmaceutics10030127 PMC6161255 30096910

[B12] Foster P. A. Woo L. W. L. Potter B. V. L. Reed M. J. Purohit A. (2008). The Use of Steroid Sulfatase Inhibitors as a Novel Therapeutic Strategy against Hormone-dependent Endometrial Cancer. Endocrinology 149 (8), 4035–4042. 10.1210/en.2008-0223 18450955 PMC2488239

[B13] Gibson D. A. Simitsidellis I. Collins F. Saunders P. T. K. (2014). Evidence of Androgen Action in Endometrial and Ovarian Cancers. Endocr. Relat. Cancer 21 (4), T203–T218. 10.1530/ERC-13-0551 24623742

[B14] Hevir N. Šinkovec J. Rižner T. L. (2011). Disturbed Expression of Phase I and Phase II Estrogen-Metabolizing Enzymes in Endometrial Cancer: Lower Levels of CYP1B1 and Increased Expression of S-COMT. Mol. Cell Endocrinol. 331 (1), 158–167. 10.1016/j.mce.2010.09.011 20887769

[B15] Hevir-Kene N. Rižner T. L. (2015). The Endometrial Cancer Cell Lines Ishikawa and HEC-1A, and the Control Cell Line HIEEC, Differ in Expression of Estrogen Biosynthetic and Metabolic Genes, and in Androstenedione and Estrone-Sulfate Metabolism. Chem. Biol. Interact. 234, 309–319. 10.1016/j.cbi.2014.11.015 25437045

[B16] Inoue M. (2001). Current Molecular Aspects of the Carcinogenesis of the Uterine Endometrium. Int. J. Gynecol. Cancer 11 (5), 339–348. 10.1046/j.1525-1438.2001.01046.x 11737463

[B17] Kandoth C. Kandoth C. Schultz N. Cherniack A. D. Akbani R. Liu Y. (2013). Integrated Genomic Characterization of Endometrial Carcinoma. Nature 497 (7447), 67–73. 10.1038/nature12113 23636398 PMC3704730

[B18] König J. Seithel A. Gradhand U. Fromm M. F. (2006). Pharmacogenomics of Human OATP Transporters. Naunyn Schmied Arch. Pharmacol. 372 (6), 432–443. 10.1007/s00210-006-0040-y 16525793

[B19] Lépine J. Audet-Walsh E. Grégoire J. Têtu B. Plante M. Ménard V. (2010). Circulating Estrogens in Endometrial Cancer Cases and Their Relationship with Tissular Expression of Key Estrogen Biosynthesis and Metabolic Pathways. J. Clin. Endocrinol. Metab. 95 (6), 2689–2698. 10.1210/jc.2010-2648 20371658

[B20] Lindemann K. Eskild A. Vatten L. J. Bray F. (2010). Endometrial Cancer Incidence Trends in Norway during 1953-2007 and Predictions for 2008-2027. Int. J. Cancer 127 (11), 2661–2668. 10.1002/ijc.25267 20162667

[B21] Lu L.-Y. Hsieh Y.-C. Liu M.-Y. Lin Y.-H. Chen C.-J. Yang Y.-S. (2008). Identification and Characterization of Two Amino Acids Critical for the Substrate Inhibition of Human Dehydroepiandrosterone Sulfotransferase (SULT2A1). Mol. Pharmacol. 73 (3), 660–668. 10.1124/mol.107.041038 18042734

[B22] Malini B. Purohit A. Ganeshapillai D. Woo L. W. Potter B. V. Reed M. J. (2000). Inhibition of Steroid Sulphatase Activity by Tricyclic Coumarin Sulphamates. J. Steroid Biochem. Mol. Biol. 75 (4-5), 253–258. 10.1016/s0960-0760(00)00178-3 11282279

[B23] Morice P. Leary A. Creutzberg C. Abu-Rustum N. Darai E. (2016). Endometrial Cancer. Lancet 387 (10023), 1094–1108. 10.1016/S0140-6736(15)00130-0 26354523

[B24] Murali R. Soslow R. A. Weigelt B. (2014). Classification of Endometrial Carcinoma: More Than Two Types. Lancet Oncol. 15 (7), e268–e278. 10.1016/s1470-2045(13)70591-6 24872110

[B25] Paterni I. Granchi C. Katzenellenbogen J. A. Minutolo F. (2014). Estrogen Receptors Alpha (ERα) and Beta (ERβ): Subtype-Selective Ligands and Clinical Potential. Steroids 90, 13–29. 10.1016/j.steroids.2014.06.012 24971815 PMC4192010

[B26] Pavlič R. Vidic S. Anko M. Knific T. Büdefeld T. Marton K. (2021). Altered Profile of E1-S Transporters in Endometrial Cancer: Lower Protein Levels of ABCG2 and OSTβ and Up-Regulation of SLCO1B3 Expression. Ijms 22 (8), 3819. 10.3390/ijms22083819 33917029 PMC8067723

[B27] Poschner S. Zehl M. Maier-Salamon A. Jäger W. (2017). Simultaneous Quantification of Estrogens, Their Precursors and Conjugated Metabolites in Human Breast Cancer Cells by LC-HRMS without Derivatization. J. Pharm. Biomed. Anal. 138, 344–350. 10.1016/j.jpba.2017.02.033 28249239

[B28] Richardson G. S. Dickersin G. R. Atkins L. MacLaughlin D. T. Raam S. Merk L. P. (1984). KLE: a Cell Line with Defective Estrogen Receptor Derived from Undifferentiated Endometrial Cancer. Gynecol. Oncol. 17 (2), 213–230. 10.1016/0090-8258(84)90080-5 6706226

[B29] Rižner T. L. Šmuc T. Rupreht R. Šinkovec J. Penning T. M. (2006). AKR1C1 and AKR1C3 May Determine Progesterone and Estrogen Ratios in Endometrial Cancer. Mol. Cell. Endocrinol. 248 (1-2), 126–135. 10.1016/j.mce.2005.10.009 16338060

[B30] Rižner T. L. Thalhammer T. Özvegy-Laczka C. (2017). The Importance of Steroid Uptake and Intracrine Action in Endometrial and Ovarian Cancers. Front. Pharmacol. 8, 346. 10.3389/fphar.2017.00346 28674494 PMC5474471

[B31] Rižner T. L. (2013). Estrogen Biosynthesis, Phase I and Phase II Metabolism, and Action in Endometrial Cancer. Mol. Cell Endocrinol. 381 (1-2), 124–139. 10.1016/j.mce.2013.07.026 23911898

[B32] Roth M. Obaidat A. Hagenbuch B. (2012). OATPs, OATs and OCTs: the Organic Anion and Cation Transporters of the SLCO and SLC22A Gene Superfamilies. Br. J. Pharmacol. 165 (5), 1260–1287. 10.1111/j.1476-5381.2011.01724.x 22013971 PMC3372714

[B33] Simitsidellis I. Saunders P. T. K. Gibson D. A. (2018). Androgens and Endometrium: New Insights and New Targets. Mol. Cell Endocrinol. 465, 48–60. 10.1016/j.mce.2017.09.022 28919297

[B34] Sinreih M. Knific T. Anko M. Hevir N. Vouk K. Jerin A. (2017). The Significance of the Sulfatase Pathway for Local Estrogen Formation in Endometrial Cancer. Front. Pharmacol. 8, 368. 10.3389/fphar.2017.00368 28690541 PMC5481366

[B35] Swasti (2018). Estrogen and Progesterone Receptors in Endometrial Cancer: Where Are We Today? Gynecol. Obstet. 8 (2), e127. 10.4172/2161-0932.1000e127

[B36] Van Nyen T. Moiola C. P. Colas E. Annibali D. Amant F. (2018). Modeling Endometrial Cancer: Past, Present, and Future. Ijms 19 (8), 2348. 10.3390/ijms19082348 PMC6121384 30096949

[B37] Vivacqua A. Bonofiglio D. Recchia A. G. Musti A. M. Picard D. Andò S. (2006). The G Protein-Coupled Receptor GPR30 Mediates the Proliferative Effects Induced by 17β-Estradiol and Hydroxytamoxifen in Endometrial Cancer Cells. Mol. Endocrinol. 20 (3), 631–646. 10.1210/me.2005-0280 16239258

[B38] Wan J. Gao Y. Zeng K. Yin Y. Zhao M. Wei J. (2016). The Levels of the Sex Hormones Are Not Different between Type 1 and Type 2 Endometrial Cancer. Sci. Rep. 6, 39744. 10.1038/srep39744 28000774 PMC5175126

[B39] Way D. L. Grosso D. S. Davis J. R. Surwit E. A. Christian C. D. (1983). Characterization of a New Human Endometrial Carcinoma (RL95-2) Established in Tissue Culture. In Vitro 19 (3 Pt 1), 147–158. 10.1007/BF02618053 6339371

[B40] Xu S. Yu S. Dong D. Lee L. T. O. (2019). G Protein-Coupled Estrogen Receptor: A Potential Therapeutic Target in Cancer. Front. Endocrinol. 10, 725. 10.3389/fendo.2019.00725 PMC6823181 31708873

[B41] Yeramian A. Moreno-Bueno G. Dolcet X. Catasus L. Abal M. Colas E. (2013). Endometrial Carcinoma: Molecular Alterations Involved in Tumor Development and Progression. Oncogene 32 (4), 403–413. 10.1038/onc.2012.76 22430211

