# Supplementary material for: In the Model Cell Lines of Moderately and Poorly Differentiated Endometrial Carcinoma, Estrogens Can Be Formed via the Sulfatase Pathway
Source: Front Mol Biosci. 2021 Nov 5;8:743403. doi: 10.3389/fmolb.2021.743403 (PMC8602794; doi:10.3389/fmolb.2021.743403)
Supplement: Supplementary file 1 [file datasheet1.docx]

In the Model Cell Lines of Moderately and Poorly Differentiated Endometrial Carcinoma, Estrogens Can Be Formed via the Sulfatase Pathway

Renata Pavlič^1^, Marija Gjorgoska^1^, Eva Hafner^1^, Maša Sinreih^1^, Kristina Gajser^1^, Stefan Poschner^2^, Walter Jäger^2^, and Tea Lanišnik Rižner^1,^*

^1^Laboratory for Molecular Basis of Hormone-Dependent Diseases and Biomarkers, Institute of Biochemistry and Molecular Genetics, Faculty of Medicine, University of Ljubljana, Ljubljana, Slovenia

^2^Department of Pharmaceutical Sciences, University of Vienna, Vienna, Austria

Supplementary Material

**Western blotting for AKR1C3, COMT and SULT1E1**

Samples of 30 μg protein were separated using SDS PAGE in 10% Tris-glycine gels (COMT and SULT1E1) and 12% Tris-glycine gels (AKR1C3), and then transferred to *poly* (vinylidene fluoride) membranes (Millipore, Billerica, MA, USA). Next, the membranes were blocked with 5% non-fat milk in TTBS for 1h (COMT) and 2h (SULT1E1 and AKR1C3) at room temperature. For COMT detection, the membranes were incubated with primary anti-COMT antibody (1:7500, ab5873, Millipore) in TTBS with 1% fat milk overnight. For SULT1E1 detection, the membranes were incubated with anti-SULT1E1 primary antibody (1:250, HPA028728, lot R28328, Sigma Aldrich Germany) in TTBS with 5% non-fat milk overnight. For AKR1C3 detection, the membranes were incubated with primary anti-AKR1C3 antibodies (1:5000; a6229, lot 025M4832V Sigma-Aldrich Chemie GmbH, Deisenhofen, Germany) in TTBS with 1% non-fat milk for 2h at room temperature. The next day, the membranes were incubated with the secondary antibodies, peroxidase conjugated goat anti-rabbit IgG + IgM (H + L), Jackson ImmunoResearch Laboratories Inc., USA; for COMT: 1:10,000, 2h at room temperature, for SULT1E1: 1:5000, 1h at 4 °C, and with peroxidase-conjugated goat anti-mouse IgG + IgM (H + L), Jackson ImmunoResearch Laboratories Inc., USA, 1:5000, for AKR1C3: 1:5000, 2h at room temperature. GAPDH (1:10,000; G8795, lot 045M4799V, Sigma Aldrich Chemie GmbH, Deisenhofen, Germany) was used as a normalization control for COMT, α-tubulin (1:10,000; ab52866, lot GR3241238-9, Abcam, Cambridge, United Kingdom) for SULT1E1, and β-actin (1:3000; A5441, Sigma-Aldrich Chemie GmbH, Deisenhofen, Germany) for AKR1C3. SuperSignal™ West Pico Plus Chemiluminescent Substrate (34579, Thermo Fischer Scientific, Waltham, MA, USA) was used for chemiluminescent detection, with a CCD camera (LAS-4000; Fujifilm, Tokyo, Japan). Differential expression of COMT, SULT1E1 and AKR1C3 was determined using the ImageJ program.


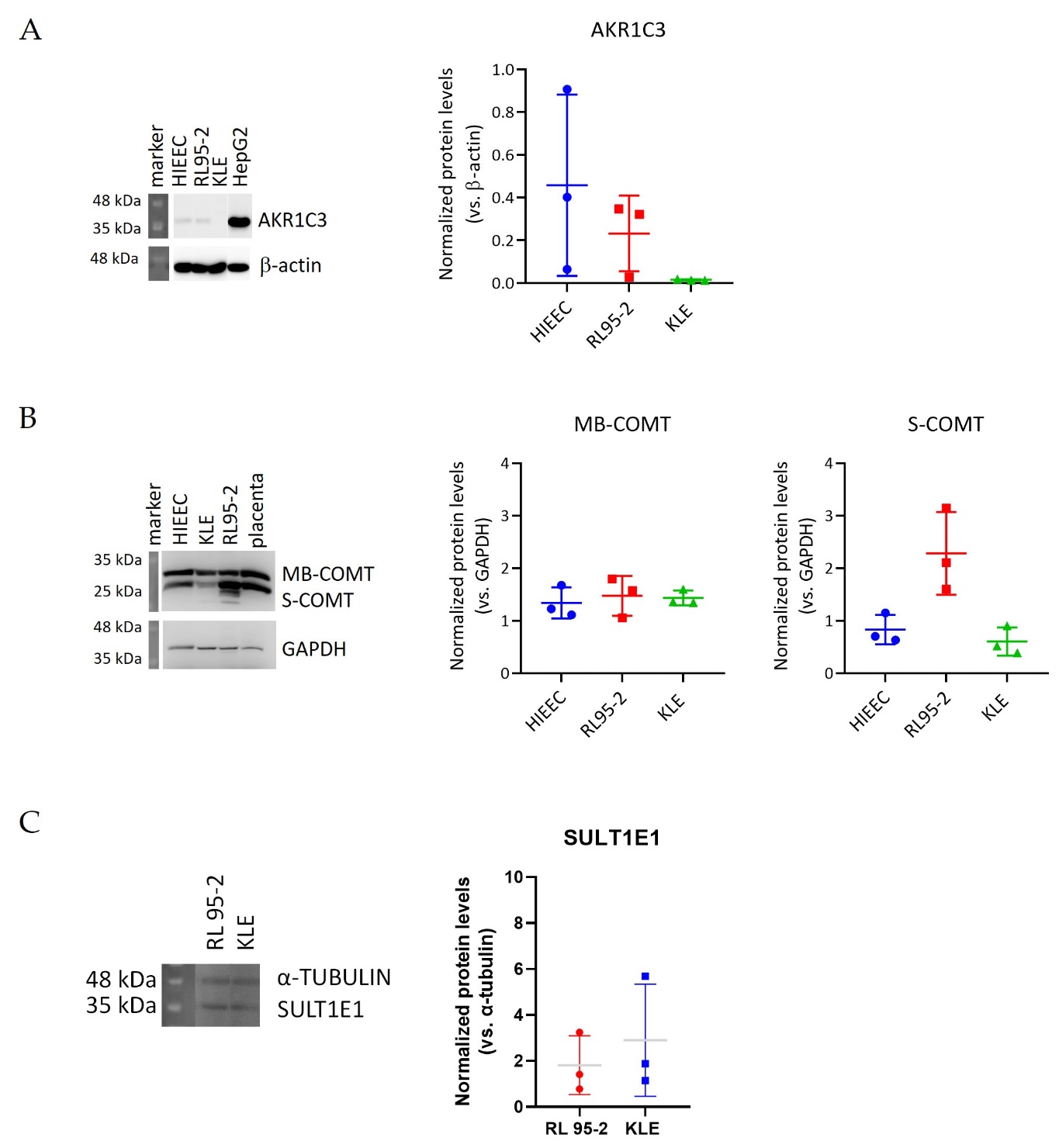


**Supplementary Figure S1.** Protein levels of AKR1C3 (**A**), membrane bound (MB) and soluble (S) COMT (**B**), and (**C**) SULT1E1 in HIEEC, RL95-2, and KLE cells. Cells HepG2 or placenta tissue samples were chosen as positive controls. Left: Representative Western blots. Right: Quantification of protein levels, normalized to GAPDH, β-actin or α-tubulin as control, with three biological replicates for each cell line. Statistical analysis: Kruskal–Wallis tests, with Dunn’s multiple comparisons. For whole Western blots and densitometry readings see Supplementary Figures 2,3,4.


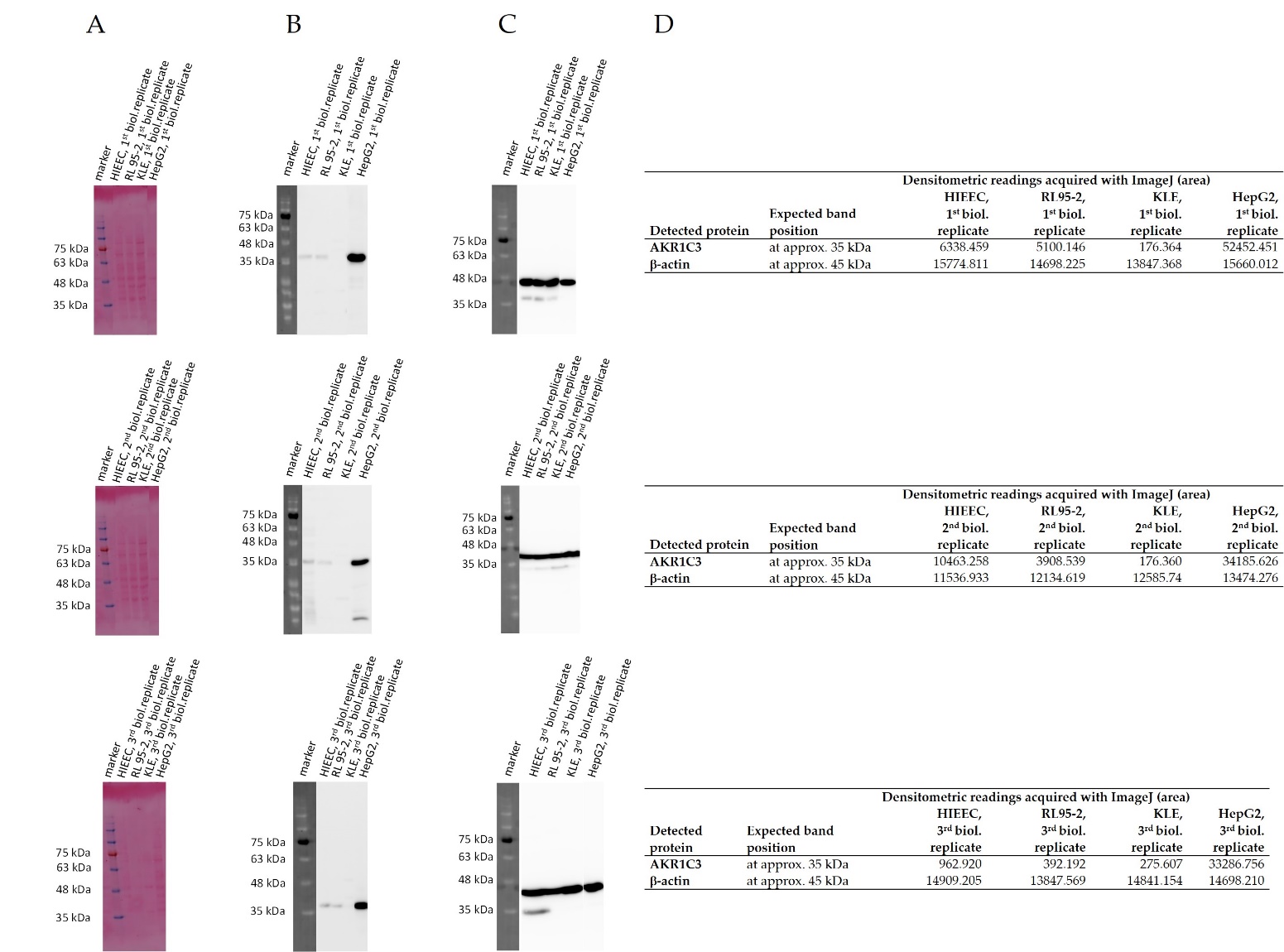


**Supplementary Figure S2.** Uncropped Western blots for detection of AKR1C3 and β-actin. A) Full-length membranes after staining with Ponceau S, B) full-length membranes after chemiluminescent detection of AKR1C3, C) full-length membranes after chemiluminescent detection of β-actin, D) densitometric readings for quantification acquired with Image J program. Membrane no. 1 was chosen as representative membrane.


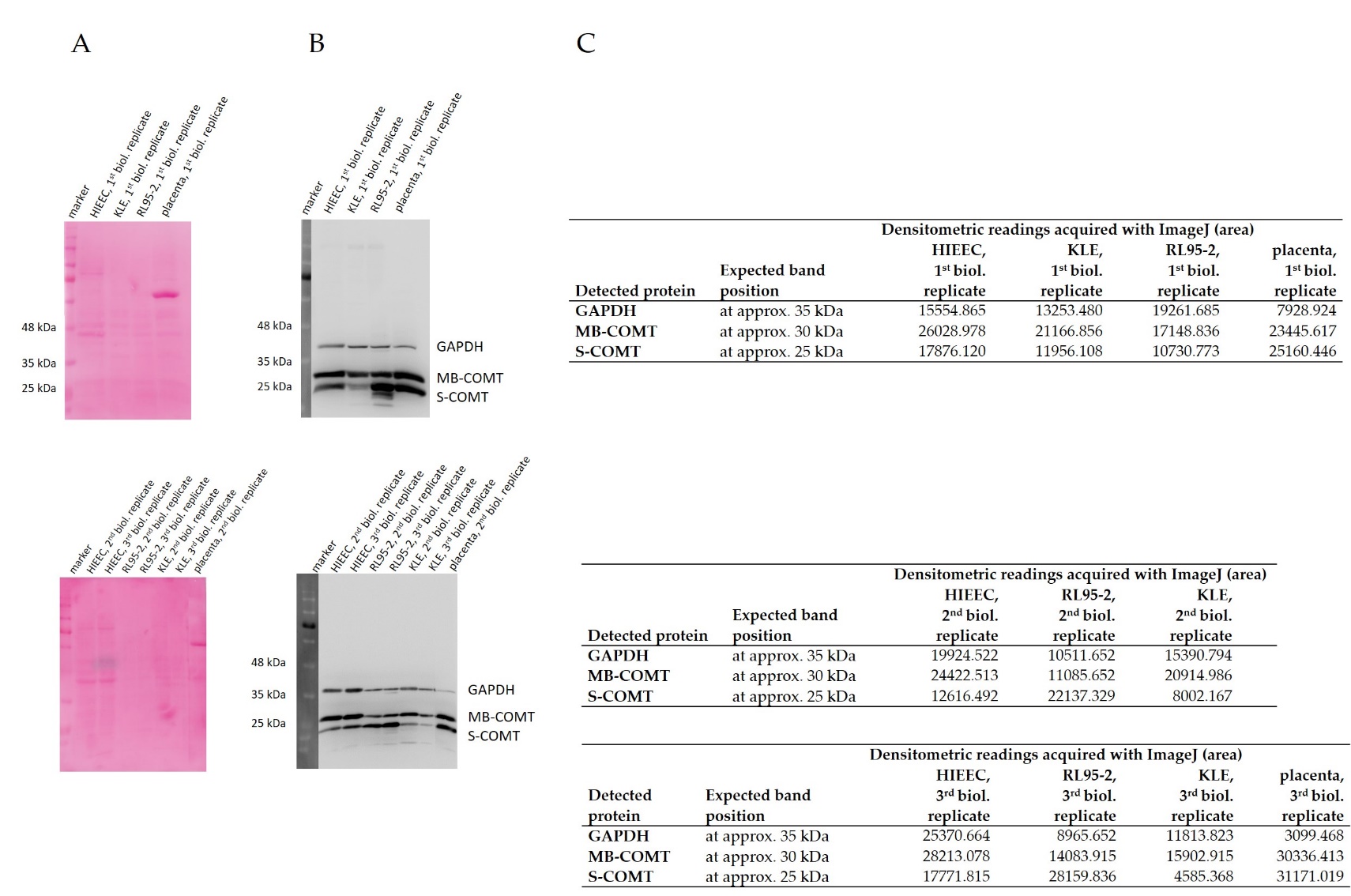


**Supplementary Figure S3.** Uncropped Western blots for detection of COMT and GAPDH. A) Full-length membranes after staining with Ponceau S, B) full-length membranes after chemiluminescent detection of membrane bound (MB)- and soluble (S)-COMT, C) full-length membranes after chemiluminescent detection of GAPDH, D) densitometric readings for quantification acquired with Image J program. Membrane no. 1 was chosen as representative membrane.


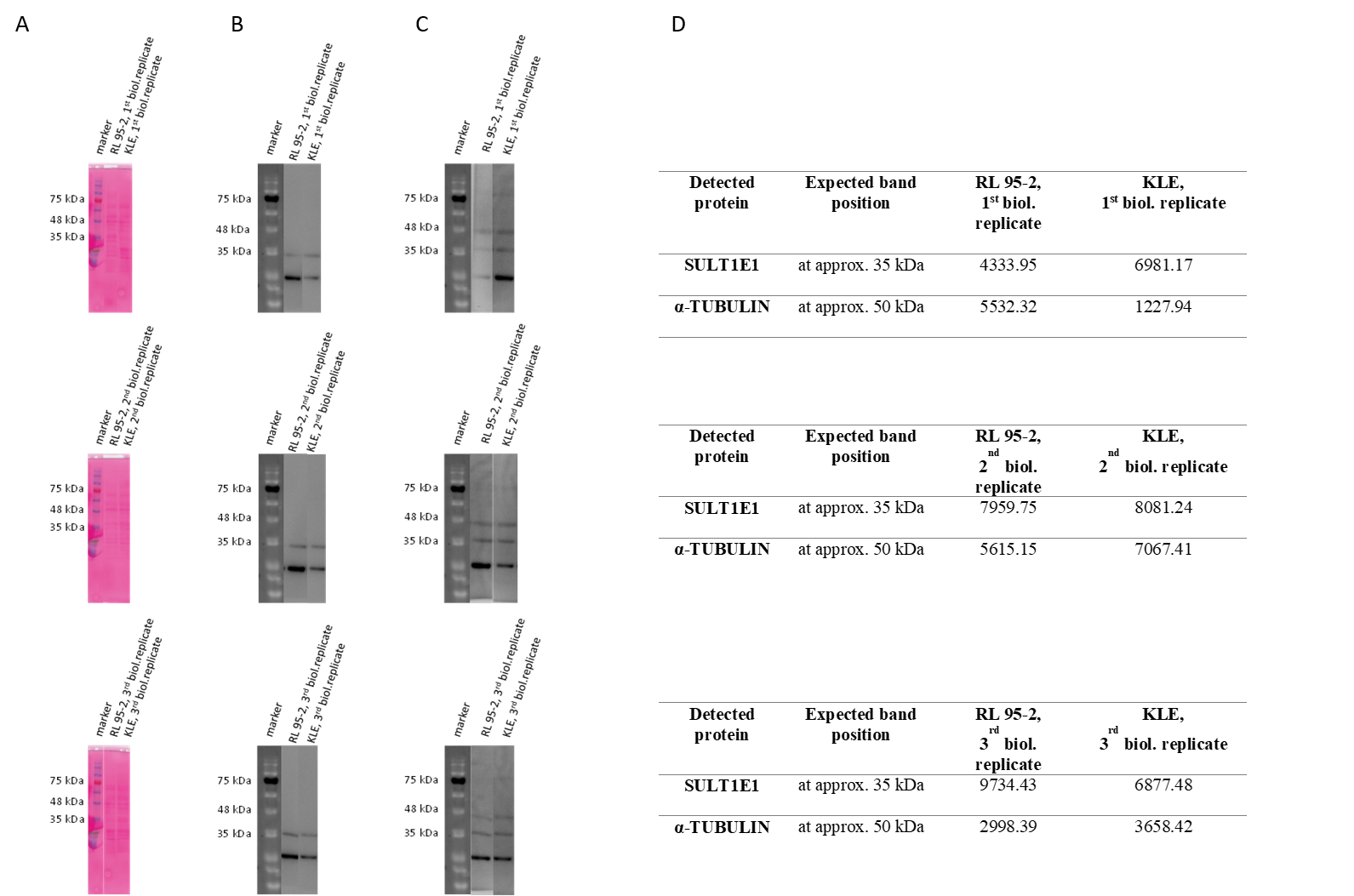


**Supplementary Figure S4.** Uncropped Western blots for detection of SULT1E1 and α-tubulin. A) Full-length membranes after staining with Ponceau S, B) full-length membranes after chemiluminescent detection of SULT1E1, C) full-length membranes after chemiluminescent detection of α-tubulin, D) densitometric readings for quantification acquired with Image J program. Membrane with 1st biological replicates was chosen as representative membrane of SULT1E1 levels in investigated cell lines.

**
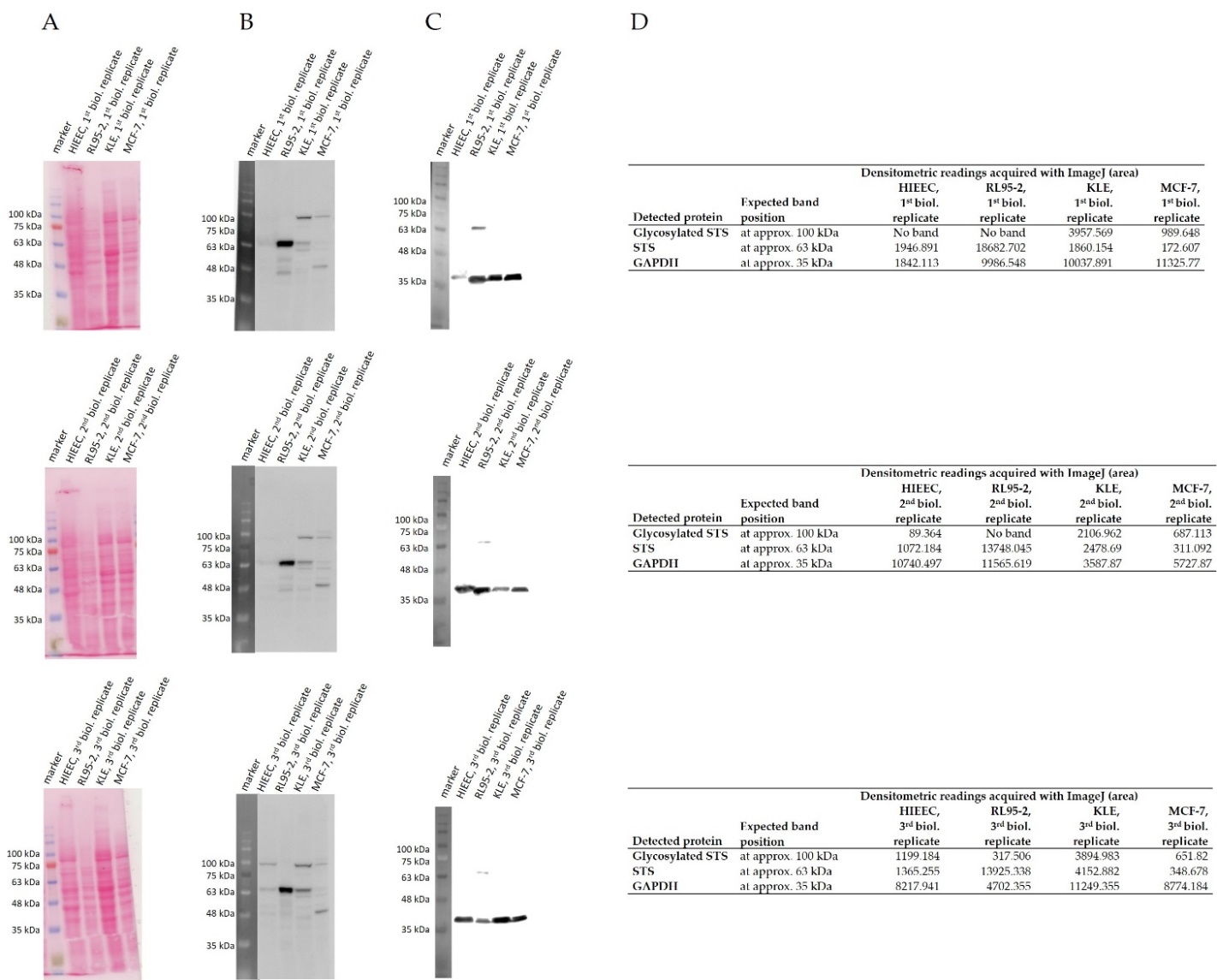
**

**Supplementary Figure S5.** Uncropped Western blots for detection of STS and GAPDH. A) Full-length membranes after staining with Ponceau S, B) full-length membranes after chemiluminescent detection of STS, C) full-length membranes after chemiluminescent detection of GAPDH, D) densitometric readings for quantification acquired with Image J program. Membrane with 2^nd^ biological replicates was chosen as representative membrane of STS levels in investigated cell lines.


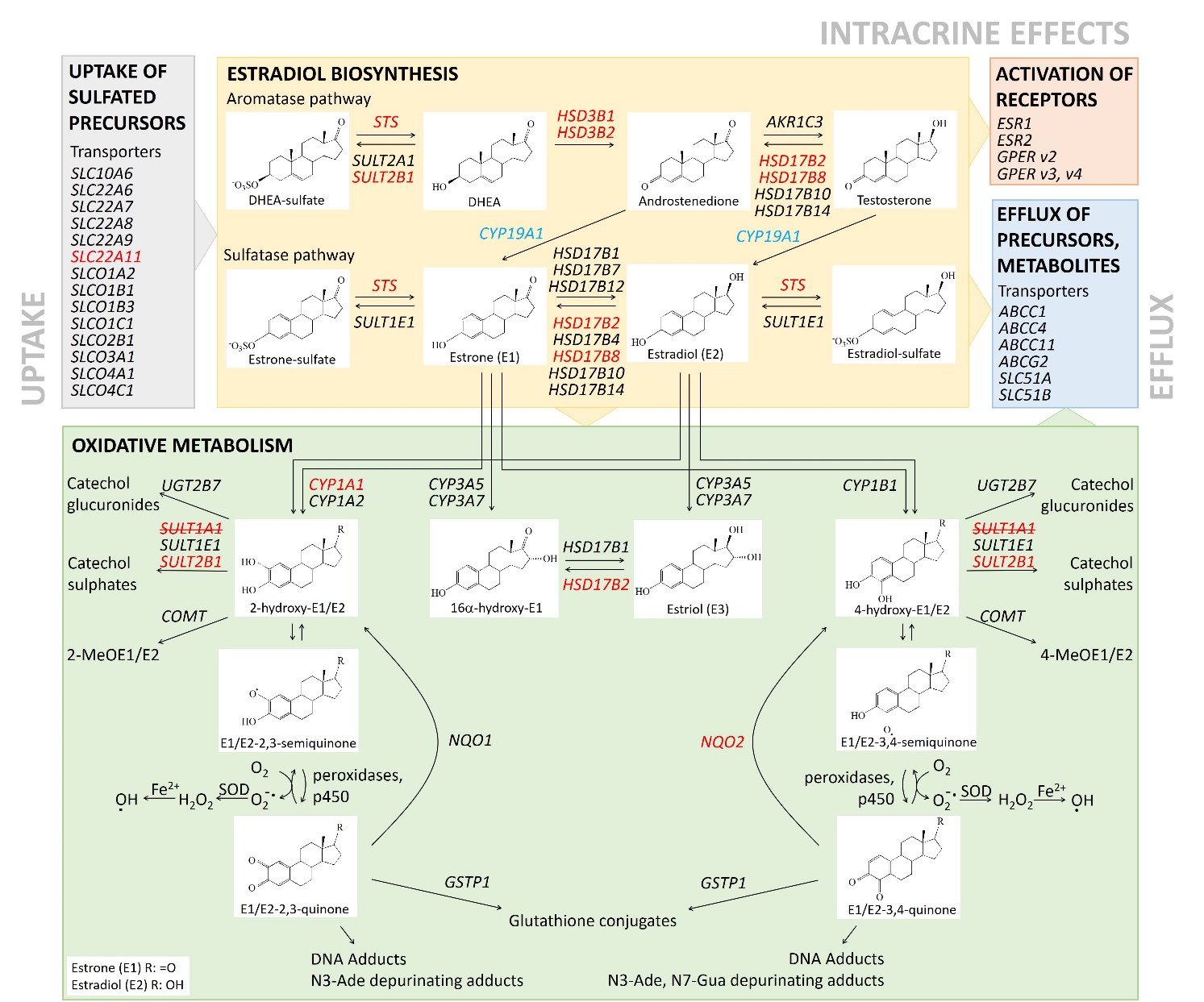


**Supplementary Figure S6.** Schematic representation of local estradiol biosynthesis and metabolism in RL95-2 and HIEEC cells. Genes with higher and lower relative expression for RL95-2 *versus* HIEEC cells are indicated in red and blue, respectively. Genes in red and blue with strikethrough were not expressed in either in HIEEC or RL95-2 cells, respectively. Data were normalized to the expression of *HPRT1*. Normalized RNA values for each of the genes are shown in Supplementary Table 3.


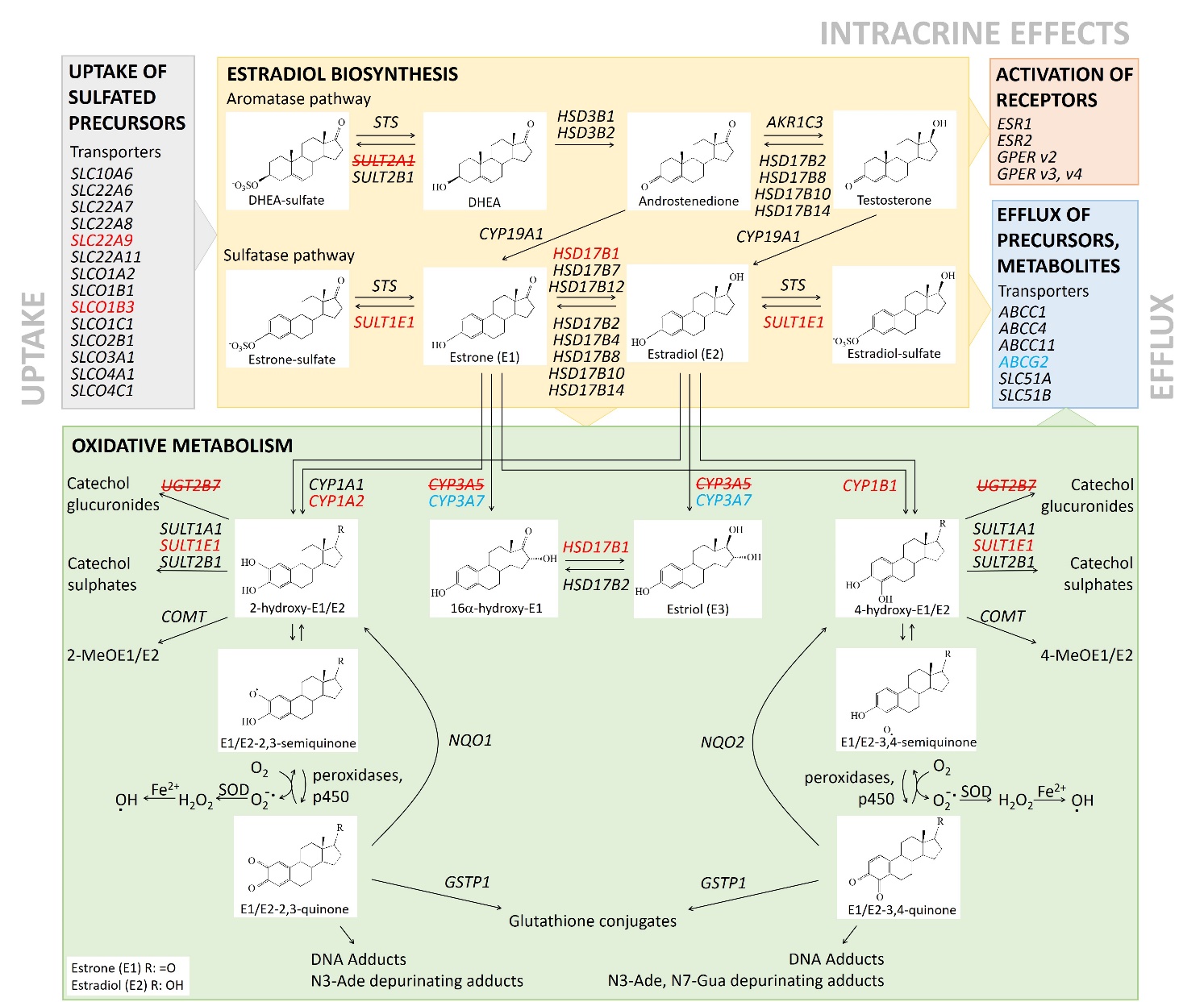


**Supplementary Figure S7.** Schematic representation of local estradiol biosynthesis and metabolism in KLE and HIEEC cells. Genes with higher and lower relative expression for KLE *versus* HIEEC are indicated in red and blue, respectively. Genes in red and blue with strikethrough were not expressed in either in HIEEC or KLE cells, respectively. The data were normalized to the expression of *HPRT1*. Normalized RNA values for each of the genes are shown in Supplementary Table 3.

**8.5 nM E1-S 85 nM E1-S**


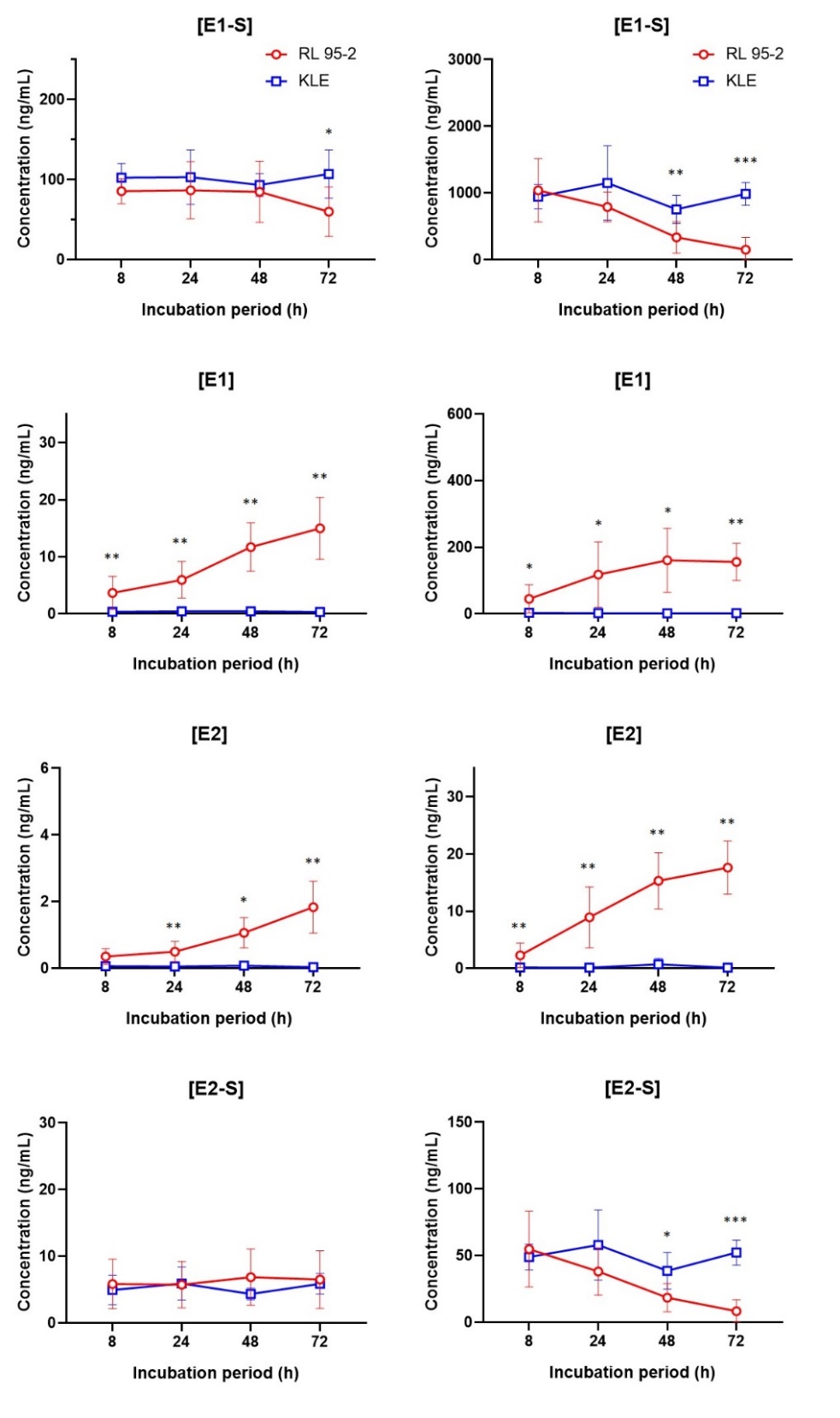


**Supplementary Figure S8.** E1-S metabolism in RL95-2 and KLE cells. Profiles of estrogen metabolites E1-S, E1, E2, E2-S after incubation with 8.5 nM E1-S (left) or 85 nM E1-S (right). Data are means ± SD. *, p<0.05; **, p<0.01 (ANOVA plus Tukey’s tests).

**Supplementary Table S1.** Selected reaction monitoring transitions and retention times for E1, E1-S, E2, E2-S, E2-d_2_.

| Steroid | Q1 (Da) | Q3 (Da) | Rt (min) |
| --- | --- | --- | --- |
| E1 (1) | 268.933 | 145.100 | 7.30 |
| E1 (2) | 268.933 | 142.900 | 7.30 |
| E1-S (1) | 349.025 | 269.000 | 6.00 |
| E1-S (2) | 349.025 | 145.100 | 6.00 |
| E2 (1) | 270.990 | 145.200 | 7.30 |
| E2 (2) | 270.990 | 183.100 | 7.30 |
| E2-S (1) | 350.901 | 270.800 | 6.00 |
| E2-S (2) | 350.901 | 145.000 | 6.00 |
| E2-d_2_ (1) | 272.990 | 147.200 | 7.30 |
| E2-d_2_ (2) | 272.990 | 185.100 | 7.30 |

E1, estrone; E1-S, estrone sulfate; E2, estradiol; E2-S, estradiol sulfate; E2-d_2_, dideuterio-estradiol. Ion transition used for quantification (1), detection (2).

**Supplementary Table S2.** Expression of genes of interest in RL95-2 and KLE cells.

| **Gene** | **RL95-2** |  |  | **KLE** |  |  | **FR** |  |
| --- | --- | --- | --- | --- | --- | --- | --- | --- |
|  | **Normalized**  **RNA x 10^13^** | **SD** |  | **Normalized**  **RNA x 10^13^** | **SD** |  | **(KLE/RL95-2)** | **p** |
| *AKR1C3* | 41745.45 | 4890.83 |  | 822.75 | 384.36 |  | ˗50.74 | * |
| *COMT* | 20327780.62 | 4009171.84 |  | 3237923.63 | 989640.59 |  | -6.28 | * |
| *CYP19A1* | 14.39 | 11.38 |  | 28.37 | 5.75 |  | 1.97 | ns |
| *CYP1A1* | 1843030.72 | 1323493.27 |  | 174546.68 | 174237.48 |  | -10.56 | * |
| *CYP1A2* | 289.38 | 170.35 |  | 2719.56 | 3585.12 |  | 9.40 | ns |
| *CYP1B1* | 777179.38 | 162196.46 |  | 13266394.84 | 4644535.86 |  | 17.07 | * |
| *CYP3A5* | 75.14 | 86.50 |  | 134.37 | 47.21 |  | 1.79 | ns |
| *CYP3A7* | 1164.74 | 1008.57 |  | 16.63 | 21.60 |  | -70.05 | * |
| *ESR1* | 119485.39 | 140309.00 |  | 182995.53 | 78200.28 |  | 1.53 | ns |
| *ESR2* | 35056.61 | 15158.05 |  | 48143.86 | 14504.63 |  | 1.37 | ns |
| *GPER v2* | 41502.62 | 11390.13 |  | 18.95 | 19.30 |  | -2189.72 | * |
| *GPER v3, v4* | 167194.69 | 68637.36 |  | 2067.99 | 935.89 |  | -80.85 | * |
| *GSTP1* | 19686829.44 | 2400950.63 |  | 9164463.03 | 1514195.06 |  | -2.15 | * |
| *HSD17B1* | 3660.05 | 1881.04 |  | 8976.33 | 5786.95 |  | 2.45 | ns |
| *HSD17B10* | 1308496.77 | 309947.91 |  | 1985169.67 | 554387.69 |  | 1.52 | ns |
| *HSD17B12* | 26033450.76 | 3146858.43 |  | 10986878.66 | 2492769.88 |  | -2.37 | * |
| *HSD17B14* | 13135.97 | 4952.30 |  | 103236.51 | 26096.05 |  | 7.86 | * |
| *HSD17B2* | 95335008.80 | 19703361.31 |  | 73125.92 | 60045.06 |  | -1303.71 | * |
| *HSD17B4* | 6817656.07 | 1307628.65 |  | 3893328.11 | 1136976.11 |  | -1.75 | * |
| *HSD17B7* | 406240.50 | 59093.54 |  | 672360.65 | 269423.38 |  | 1.66 | ns |
| *HSD17B8* | 557235.94 | 103527.53 |  | 341908.38 | 250014.15 |  | -1.63 | ns |
| *HSD3B1* | 2122.68 | 1203.99 |  | 27.87 | 24.98 |  | -76.18 | * |
| *HSD3B2* | 265.06 | 200.97 |  | 59.16 | 9.01 |  | -4.48 | * |
| *NQO1* | 87466781.89 | 11371034.28 |  | 6891257.22 | 1965964.16 |  | -12.69 | * |
| *NQO2* | 1369863.54 | 418636.77 |  | 208715.01 | 64910.36 |  | -6.56 | * |
| *STS* | 21834067.31 | 7518111.47 |  | 2360208.59 | 335060.13 |  | -9.25 | * |
| *SULT1A1* | 2954.56 | 470.33 |  | 0.00 | 0.00 |  |  | * |
| *SULT1E1* | 6761.94 | 3774.30 |  | 92232.71 | 16431.03 |  | 13.64 | * |
| *SULT2A1* | 0.00 | 0.00 |  | 72.44 | 20.85 |  |  | * |
| *SULT2B1* | 57701.07 | 37069.24 |  | 3255.98 | 709.09 |  | -17.72 | * |
| *UGT2B7* | 0.00 | 0.00 |  | 60.73 | 25.79 |  |  | * |
| *ABCC1* | 1012069.12 | 727492.18 |  | 1339510.38 | 907590.15 |  | 1.32 | ns |
| *ABCC11* | 0.00 | 0.00 |  | 0.00 | 0.00 |  |  | ns |
| *ABCC4* | 73610.26 | 55632.51 |  | 48441.76 | 34712.41 |  | -1.52 | ns |
| *ABCG2* | 37067.35 | 39642.37 |  | 21850.45 | 11121.17 |  | -1.70 | ns |
| *SLC10A6* | 502.19 | 244.06 |  | 2750.57 | 527.17 |  | 5.48 | * |
| *SLC22A11* | 359714.69 | 282910.54 |  | 6068.62 | 3105.33 |  | -59.27 | * |
| *SLC22A6* | 891.45 | 781.74 |  | 74.92 | 69.19 |  | -11.90 | ns |
| *SLC22A7* | 13322.49 | 15225.24 |  | 850.53 | 795.66 |  | -15.66 | ns |
| *SLC22A8* | 34741.95 | 48932.77 |  | 430.60 | 252.01 |  | -80.68 | ns |
| *SLC22A9* | 21007.39 | 24017.42 |  | 104430.79 | 69900.42 |  | 4.97 | * |
| *SLC51A* | 23081.63 | 25365.31 |  | 22418.61 | 8687.17 |  | -1.03 | ns |
| *SLC51B* | 3851.52 | 4413.14 |  | 5545.13 | 2847.47 |  | 1.44 | ns |
| *SLCO1A2* | 14.67 | 12.35 |  | 50362.87 | 70773.76 |  | 3433.55 | * |
| *SLCO1B1* | 299.91 | 597.99 |  | 93.43 | 162.83 |  | -3.21 | ns |
| *SLCO1B3* | 672.93 | 287.70 |  | 1548744.84 | 740360.22 |  | 2301.50 | * |
| *SLCO1C1* | 2.81 | 5.63 |  | 1072.89 | 1010.77 |  | 381.23 | * |
| *SLCO2B1* | 38251.34 | 47516.98 |  | 1330.95 | 751.63 |  | -28.74 | ns |
| *SLCO3A1* | 770.62 | 403.21 |  | 14658.10 | 11480.19 |  | 19.02 | * |
| *SLCO4A1* | 200331.14 | 162548.73 |  | 59741.79 | 13216.67 |  | -3.35 | * |
| *SLCO4C1* | 16306.11 | 19290.05 |  | 61136.53 | 24002.97 |  | 3.75 | ns |

FR – fold regulation. Statistical analysis: Mann Whitney test. ns, not significant; *, p<0.05.

**Supplementary Table S3.** Expression of genes of interest in HIEEC, RL95-2 and KLE cells, normalized to *HPRT1.* To remove inter-plate variability in comparison of RL95-2 or KLE with HIEEC cells only genes with 10-fold or higher significant differences in expression were considered important in data interpretation (highlighted orange).

|  | **HIEEC** |  |  | **RL95-2** |  |  | **KLE** |  |  | **RL95-2/**  **HIEEC** |  |  | **KLE/**  **HIEEC** |  |
| --- | --- | --- | --- | --- | --- | --- | --- | --- | --- | --- | --- | --- | --- | --- |
| **Gene** | **Normalized RNA** | **SD** |  | **Normalized RNA** | **SD** |  | **Normalized RNA** | **SD** |  | **FR** | **p** |  | **FR** | **p** |
| *AKR1C3* | 1.1535E-09 | 2.8332E-10 |  | 4.9152E-09 | 8.3311E-10 |  | 6.2228E-11 | 2.3707E-11 |  | 4.26 | ns |  | -18.54 | ns |
| *COMT* | 2.7183E-07 | 9.2011E-08 |  | 2.3930E-06 | 5.7542E-07 |  | 2.4076E-07 | 4.1834E-08 |  | 8.80 | ns |  | -1.13 | ns |
| *CYP19A1* | 2.7932E-11 | 7.2548E-12 |  | 1.7359E-12 | 1.3675E-12 |  | 2.3678E-12 | 1.3392E-12 |  | -16.09 | * |  | -11.80 | ns |
| *CYP1A1* | 1.9276E-09 | 4.7090E-10 |  | 2.4601E-07 | 2.3280E-07 |  | 1.2372E-08 | 1.1449E-08 |  | 127.63 | * |  | 6.42 | ns |
| *CYP1A2* | 2.2227E-12 | 2.0329E-12 |  | 3.7009E-11 | 3.0251E-11 |  | 1.9379E-10 | 2.0808E-10 |  | 16.65 | ns |  | 87.19 | * |
| *CYP1B1* | 1.2344E-08 | 3.6888E-09 |  | 9.3946E-08 | 3.6969E-08 |  | 1.0017E-06 | 2.7886E-07 |  | 7.61 | ns |  | 81.14 | ** |
| *CYP3A5* | 0.0000E+00 | 0.0000E+00 |  | 1.0779E-11 | 1.4056E-11 |  | 1.0566E-11 | 4.3892E-12 |  |  | ns |  |  | * |
| *CYP3A7* | 1.0270E-10 | 8.5361E-12 |  | 1.5536E-10 | 1.7233E-10 |  | 1.1025E-12 | 1.4340E-12 |  | 1.51 | ns |  | -93.16 | * |
| *ESR1* | 4.5710E-09 | 2.5779E-10 |  | 1.2621E-08 | 1.2005E-08 |  | 1.4127E-08 | 6.0644E-09 |  | 2.76 | ns |  | 3.09 | ns |
| *ESR2* | 6.9823E-11 | 4.8277E-11 |  | 4.4995E-09 | 2.9984E-09 |  | 3.8467E-09 | 1.7948E-09 |  | 64.44 | ns |  | 55.09 | ns |
| *GPER v2* | 9.5736E-10 | 2.8373E-10 |  | 4.8198E-09 | 9.5436E-10 |  | 1.6907E-12 | 1.8012E-12 |  | 5.03 | ns |  | -566.24 | ns |
| *GPER v3, v4* | 2.0571E-10 | 3.1324E-11 |  | 1.9336E-08 | 7.3223E-09 |  | 1.6048E-10 | 7.3622E-11 |  | 94.00 | ns |  | -1.28 | ns |
| *GSTP1* | 3.0963E-06 | 4.3585E-07 |  | 2.3450E-06 | 6.1656E-07 |  | 7.0309E-07 | 1.3660E-07 |  | -1.32 | ns |  | -4.40 | * |
| *HSD17B1* | 4.0190E-12 | 4.0534E-12 |  | 4.6766E-10 | 3.3682E-10 |  | 8.2247E-10 | 8.3430E-10 |  | 116.36 | ns |  | 204.64 | * |
| *HSD17B10* | 1.7355E-07 | 3.6107E-08 |  | 1.5807E-07 | 5.4966E-08 |  | 1.5219E-07 | 4.5263E-08 |  | -1.10 | ns |  | -1.14 | ns |
| *HSD17B12* | 5.6482E-07 | 2.1330E-08 |  | 3.1382E-06 | 1.0718E-06 |  | 8.2221E-07 | 8.5118E-08 |  | 5.56 | ** |  | 1.46 | ns |
| *HSD17B14* | 7.0138E-09 | 2.2176E-09 |  | 1.6518E-09 | 1.0176E-09 |  | 7.8905E-09 | 2.0369E-09 |  | -4.25 | ns |  | 1.12 | ns |
| *HSD17B2* | 2.5862E-10 | 1.9547E-10 |  | 1.1136E-05 | 2.0065E-06 |  | 5.1769E-09 | 3.1465E-09 |  | 43060.24 | ** |  | 20.02 | ns |
| *HSD17B4* | 2.3579E-07 | 5.7219E-08 |  | 8.1533E-07 | 2.2382E-07 |  | 2.8552E-07 | 2.6992E-09 |  | 3.46 | * |  | 1.21 | ns |
| *HSD17B7* | 1.3765E-08 | 1.4497E-09 |  | 4.9192E-08 | 1.7312E-08 |  | 5.0498E-08 | 1.6020E-08 |  | 3.57 | ns |  | 3.67 | ns |
| *HSD17B8* | 1.0257E-09 | 7.9551E-11 |  | 6.7040E-08 | 2.0825E-08 |  | 2.4524E-08 | 1.5032E-08 |  | 65.36 | ** |  | 23.91 | ns |
| *HSD3B1* | 1.3140E-12 | 2.2760E-12 |  | 2.7461E-10 | 2.1815E-10 |  | 2.1376E-12 | 1.6861E-12 |  | 208.99 | * |  | 1.63 | ns |
| *HSD3B2* | 1.8078E-12 | 1.6834E-12 |  | 3.5949E-11 | 3.5096E-11 |  | 4.7316E-12 | 1.8775E-12 |  | 19.89 | ** |  | 2.62 | ns |
| *NQO1* | 6.3495E-07 | 1.1689E-07 |  | 1.0259E-05 | 1.7796E-06 |  | 5.2007E-07 | 1.1536E-07 |  | 16.16 | ns |  | -1.22 | ns |
| *NQO2* | 1.2276E-08 | 2.0059E-09 |  | 1.7313E-07 | 9.1361E-08 |  | 1.5481E-08 | 2.5192E-09 |  | 14.10 | ** |  | 1.26 | ns |
| *STS* | 3.6023E-08 | 4.2740E-09 |  | 2.6319E-06 | 1.2424E-06 |  | 1.8375E-07 | 5.5086E-08 |  | 73.06 | ** |  | 5.10 | ns |
| *SULT1A1* | 0.0000E+00 | 0.0000E+00 |  | 3.5364E-10 | 1.0392E-10 |  | 0.0000E+00 | 0.0000E+00 |  |  | * |  |  | ns |
| *SULT1E1* | 6.9388E-12 | 3.6645E-12 |  | 7.4724E-10 | 2.3487E-10 |  | 7.0824E-09 | 1.5716E-09 |  | 107.69 | ns |  | 1020.70 | ** |
| *SULT2A1* | 0.0000E+00 | 0.0000E+00 |  | 0.0000E+00 | 0.0000E+00 |  | 5.6678E-12 | 2.1048E-12 |  |  | ns |  |  | * |
| *SULT2B1* | 4.1286E-11 | 3.2507E-12 |  | 7.6691E-09 | 6.6692E-09 |  | 2.5005E-10 | 6.3104E-11 |  | 185.76 | ** |  | 6.06 | ns |
| *UGT2B7* | 0.0000E+00 | 0.0000E+00 |  | 0.0000E+00 | 0.0000E+00 |  | 4.5525E-12 | 1.3088E-12 |  |  | ns |  |  | * |
| *ABCC1* | 1.3298E-07 | 6.7591E-08 |  | 1.9810E-07 | 1.4231E-07 |  | 1.6696E-07 | 1.1349E-07 |  | 1.49 | ns |  | 1.26 | ns |
| *ABCC11* | 2.1197E-09 | 2.3338E-09 |  | 0.0000E+00 | 0.0000E+00 |  | 0.0000E+00 | 0.0000E+00 |  |  | ns |  |  | ns |
| *ABCC4* | 1.2157E-08 | 3.2485E-09 |  | 1.4546E-08 | 1.1431E-08 |  | 5.9755E-09 | 4.2059E-09 |  | 1.20 | ns |  | -2.03 | ns |
| *ABCG2* | 2.9859E-08 | 5.9440E-09 |  | 8.1819E-09 | 8.9350E-09 |  | 2.5187E-09 | 1.0328E-09 |  | -3.65 | * |  | -11.85 | * |
| *SLC10A6* | 4.0958E-10 | 3.9309E-11 |  | 1.0938E-10 | 6.6149E-11 |  | 3.3036E-10 | 7.5459E-11 |  | -3.74 | ** |  | -1.24 | ns |
| *SLC22A11* | 6.5716E-11 | 1.2955E-11 |  | 7.8844E-08 | 6.9739E-08 |  | 7.0892E-10 | 3.7096E-10 |  | 1199.78 | ** |  | 10.79 | ns |
| *SLC22A6* | 2.6172E-12 | 2.2749E-12 |  | 1.9525E-10 | 1.6910E-10 |  | 8.2174E-12 | 7.2833E-12 |  | 74.60 | ns |  | 3.14 | ns |
| *SLC22A7* | 1.8135E-11 | 1.9100E-11 |  | 2.9906E-09 | 3.4742E-09 |  | 8.9051E-11 | 7.9922E-11 |  | 164.91 | ns |  | 4.91 | ns |
| *SLC22A8* | 4.1317E-11 | 3.7104E-11 |  | 6.3446E-09 | 8.0790E-09 |  | 4.7569E-11 | 2.4729E-11 |  | 153.56 | ns |  | 1.15 | ns |
| *SLC22A9* | 3.2792E-11 | 2.8409E-11 |  | 4.6821E-09 | 5.3838E-09 |  | 1.2472E-08 | 8.6762E-09 |  | 142.78 | ns |  | 380.34 | * |
| *SLC51A* | 9.2395E-09 | 4.7526E-09 |  | 5.2274E-09 | 6.3286E-09 |  | 2.6440E-09 | 9.2178E-10 |  | -1.77 | ns |  | -3.49 | ns |
| *SLC51B* | 1.0370E-09 | 1.8489E-10 |  | 8.4376E-10 | 9.5112E-10 |  | 6.4827E-10 | 3.1215E-10 |  | -1.23 | ns |  | -1.60 | ns |
| *SLCO1A2* | 2.3495E-10 | 8.0529E-11 |  | 2.9432E-12 | 2.5297E-12 |  | 5.9453E-09 | 8.2450E-09 |  | -79.83 | ns |  | 25.30 | ns |
| *SLCO1B1* | 9.1510E-12 | 4.9264E-12 |  | 7.5802E-11 | 1.5116E-10 |  | 1.0197E-11 | 1.7943E-11 |  | 8.28 | ns |  | 1.11 | ns |
| *SLCO1B3* | 2.4320E-10 | 3.4292E-10 |  | 1.3632E-10 | 4.6245E-11 |  | 1.8358E-07 | 8.5332E-08 |  | -1.78 | ns |  | 754.85 | * |
| *SLCO1C1* | 2.6847E-11 | 7.9177E-12 |  | 6.2419E-13 | 1.2484E-12 |  | 1.3364E-10 | 1.3658E-10 |  | -43.01 | ns |  | 4.98 | ns |
| *SLCO2B1* | 1.1824E-09 | 2.7043E-10 |  | 8.1522E-09 | 9.7660E-09 |  | 1.5056E-10 | 7.2467E-11 |  | 6.89 | ns |  | -7.85 | * |
| *SLCO3A1* | 1.0562E-09 | 8.6734E-10 |  | 1.5711E-10 | 8.0547E-11 |  | 1.7647E-09 | 1.5396E-09 |  | -6.72 | ns |  | 1.67 | ns |
| *SLCO4A1* | 1.6218E-02 | 3.6266E-02 |  | 4.3499E-08 | 4.0782E-08 |  | 7.0916E-09 | 1.7980E-09 |  | -372846.48 | ns |  | -2287001.85 | ns |
| *SLCO4C1* | 3.2201E-02 | 4.4095E-02 |  | 3.5601E-09 | 4.1133E-09 |  | 7.4587E-09 | 3.1676E-09 |  | -9044950.46 | ns |  | -4317215.76 | ns |

FR – fold regulation. Statistical analysis: Kruskal-Wallis with Dunn’s multiple comparisons test. ns, not significant; *, p<0.05; **, p<0.01.

**Supplementary Table S4.** Expression of *ESR1* and *ESR2* in RL95-2, KLE and HIEEC cell lines.

|  | **RL95-2** |  |  | **KLE** |  |  | **HIEEC** |  |
| --- | --- | --- | --- | --- | --- | --- | --- | --- |
| **Gene** | **Normalized**  **RNA x 10^13^** | **SD** |  | **Normalized**  **RNA x 10^13^** | **SD** |  | **Normalized**  **RNA x 10^13^** | **SD** |
| *ESR1* | 119485.39 | 140309.00 |  | 182995.53 | 78200.28 |  | 20477.99* | 2237.78* |
| *ESR2* | 35056.61 | 15158.05 |  | 48143.86 | 14504.63 |  | 304.11* | 192.90* |
| *ESR1/ESR2* | 3.41 |  |  | 3.80 |  |  | 67.34 |  |

* data from previously published study (Hevir-Kene and Rižner, 2015)
